# High‐affinity iron uptake is required for optimal *Epichloë festucae* colonization of *Lolium perenne* and seed transmission

**DOI:** 10.1111/mpp.13379

**Published:** 2023-07-21

**Authors:** Wei Zhang, Natasha T. Forester, Emma R. Applegate, Xinqi Liu, Linda J. Johnson

**Affiliations:** ^1^ AgResearch Limited, Grasslands Research Centre Palmerston North New Zealand

**Keywords:** endophyte, iron, perennial ryegrass, siderophore, symbiosis, vertical transmission

## Abstract

*Epichloë festucae* uses a siderophore‐mediated system to acquire iron, which is important to maintain endophyte–grass symbioses. Here we investigate the roles of the alternative iron acquisition system, reductive iron assimilation (RIA), via disruption of the *fetC* gene, which encodes a multicopper ferroxidase, either alone (i.e., Δ*fetC*) or in combination with disruption of the gene *sidA*, which encodes a siderophore biosynthesis enzyme (i.e., Δ*fetC*/Δ*sidA*). The phenotypic characteristics of these mutants were compared to Δ*sidA* and wild‐type (WT) strains during growth under axenic culture conditions (in culture) and in symbiosis with the host grass, perennial ryegrass (in planta). Under iron deficiency, the colony growth rate of Δ*fetC* was slightly slower than that of WT, while the growth of Δ*sidA* and Δ*fetC*/Δ*sidA* mutants was severely suppressed. Siderophore analyses indicated that Δ*fetC* mutants hyperaccumulate ferriepichloënin A (FEA) at low iron concentrations and ferricrocin and FEA at higher iron concentrations. When compared to WT, all mutant strains displayed hyperbranching hyphal structures and a reduced ratio of *Epichloë* DNA to total DNA in planta. Furthermore, host colonization and vertical transmission through infection of the host seed were significantly reduced in the Δ*fetC*/Δ*sidA* mutants, confirming that high‐affinity iron uptake is a critical process for *Epichloë* transmission. Thus, RIA and siderophore iron uptake are complementary systems required for the maintenance of iron metabolism, fungal growth, and symbiosis between *E. festucae* and perennial ryegrass.

## Graphical abstract

In *Epichloë festucae*, both reductive iron assimilation and siderophore iron uptake are required for the maintenance of iron metabolism, fungal growth, and its symbiotic relationship with perennial ryegrass.

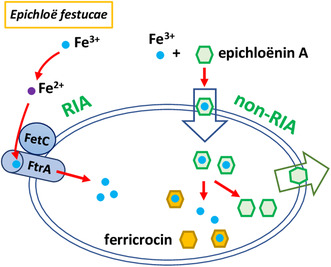

## 1 INTRODUCTION

Iron is a micronutrient that is required as a cofactor involved in a range of key biological activities, including DNA synthesis, respiration in almost all species, and photosynthesis in plants, due to its capacity to acquire and lose electrons (Guerinot & Yi, 1994; Kermeur et al., 2023; Zhang et al., 2019). Although iron is plentiful in nature, it has poor bioavailability because it is often present in the form of insoluble oxides. There are two major oxidation states for iron: ferrous (Fe^2+^) and ferric (Fe^3+^). Iron excess may harm tissues and cause diseases by acting as a redox catalyst in cell‐damaging reactive oxygen‐generating processes (Sutton & Winterbourn, 1989). To maintain iron homeostasis, fungi have evolved a variety of iron acquisition and storage systems as they lack iron excretion systems (Sørensen et al., 2014).

Iron acquisition methods for fungi fall into two broad categories: high‐affinity and low‐affinity, which serve as the primary iron assimilation systems in iron‐limited and iron‐abundant environments, respectively (Hause et al., 2002). When iron is scarce, most fungi rely on one or two high‐affinity iron acquisition processes to gather sufficient iron from the plant, the animal, or the environment. One approach is based on the secretion, by microorganisms, of small, high‐affinity Fe^3+^‐chelating compounds called siderophores. These siderophores act as carriers of iron across cell membranes (Haas, 2003; Liu et al., 2021). Extracellular siderophores aid in iron absorption, while intracellular siderophores are involved in iron storage, sequestration, and iron transportation (Forester et al., 2018; Johnson et al., 2013). Extracellular siderophores bind extracellular Fe^3+^, and ferrisiderophores are subsequently transported into the cell through specialized transport mechanisms and Fe^3+^ is released; excess Fe^3+^ may be retained by intracellular siderophores (Stintzi et al., 2000). The alternative route for iron uptake is via reductive iron assimilation (RIA), for example, in *Saccharomyces cerevisiae*, which uses a multicopper ferroxidase enzyme, Fet3p (orthologous to FetC in this study), which receives Fe^2+^ from cell surface iron reductases such as Fre1p and Fre2p and then transfers Fe^3+^ to the iron permease Ftr1p. FetC is a type I membrane protein with a single transmembrane domain at its C‐terminus and contains three distinct copper sites previously identified in copper proteins (Solomon et al., 1996). RIA requires the complex of Fet3p and Ftr1p assembled in the endoplasmic reticulum to achieve proper cell surface targeting (Kosman, 2003).

Iron absorption and metabolism is not only essential for normal biological functions but also contributes to the pathogenicity of both bacterial (Expert, 1999; Taguchi et al., 2010) and fungal plant disease agents (Eichhorn et al., 2006; Greenshields et al., 2007; Oide et al., 2006). Certain fungi, such as *Aspergillus fumigatus* (Hissen et al., 2005), *Cochliobolus heterostrophus* (Condon et al., 2014), *Nomuraea rileyi* (Li et al., 2016), *Schizosaccharomyces pombe* (Labbé et al., 2007), and *Ustilago maydis* (Mei et al., 1993), have evolved both non‐RIA (siderophore‐mediated) and RIA systems to acquire iron in low‐iron environments. The pathogenic fungi need at least one system to induce virulence. For example, only siderophores are required for the virulence of *A. fumigatus*, a major airborne fungal infection agent of humans (Schrettl et al., 2004), and *Nomuraea rileyi*, a dimorphic insect pathogen (Li et al., 2016). In comparison, only RIA is required for the virulence of *Ustilago maydis*, a smut pathogen that infects maize (*Zea mays*) (Eichhorn et al., 2006). Moreover, both siderophores and RIA are required for the full virulence of *Colletotrichum graminicola* and *C. heterostrophus*, both of which are pathogens that infect maize (Albarouki et al., 2014; Albarouki & Deising, 2013; Condon et al., 2014).

*Epichloë* species (family *Clavicipitaceae*) are filamentous fungal endophytes that form persistent, mutualistic, and host‐specific relationships with *Pooideae* cool season grasses (family *Poaceae*). *Epichloë festucae* dwells in the intercellular spaces (apoplast) of plant aerial tissues, where it acquires nutrients from the host plant (Christensen et al., 2002; Sattelmacher, 2001). In *E. festucae*, two hydroxamate siderophores have been characterized in culture: secreted epichloënin A (EA, ferrated form is ferriepichloënin A [FEA]) and intracellular ferricrocin (FC) (Forester et al., 2018; Johnson et al., 2013). A key early biosynthesis enzyme, *N* ^5^‐hydroxy‐l‐ornithine monooxygenase, encoded by the *sidA* gene, is required to produce both siderophores. Subsequently, two nonribosomal peptide synthetases, SidN (siderophore synthetase) and SidC (siderophore synthetase for FC), assemble EA and FC, respectively, from ornithine‐derived intermediates. Hence, in the *E. festucae* mutant strains Δ*sidN* and ∆*sidC*, the production of EA and FC, respectively, is abolished, while in Δ*sidA*, siderophore production is abolished (Forester et al., 2018). Aberrant hyphal growth is a phenotype of these mutants that is observed under iron‐deficient conditions (Forester et al., 2018). However, the cultures are still viable, presumably due to the presence of the complementary RIA system, but the degree of importance of RIA has yet to be clarified for *E. festucae*, particularly during symbiosis. Johnson et al. (2013) described the presence of two putative orthologues to RIA component genes, *fetC* (GenBank accession JN132406.1) and *ftrA* (GenBank accession JN132405.1), in the genome of *E. festucae* strain Fl1; the expression of these genes was controlled by a shared bidirectional promoter. The current evidence suggests that both genes are required for a functioning RIA system (Albarouki & Deising, 2013; Condon et al., 2014; Kosman, 2003); hence, *fetC* was chosen for further study. This study aims to elucidate (1) the functions of the RIA system in *E. festucae* in culture and in planta and (2) the contribution of each iron uptake system to vertical endophyte transmission, a critical component for the persistence of this symbiosis.

## 2 RESULTS

### 2.1 Inactivation of high‐affinity iron uptake systems

This study characterized the role of RIA by disrupting the multicopper ferroxidase gene (∆*fetC*) and by abolishing both RIA and siderophore‐mediated iron uptake (∆*fetC*/∆*sidA*). The mutants were characterized in culture and in planta and were compared to the previously characterized siderophore mutant Δ*sidA* (Forester et al., 2018). Table S1 provides plasmids and biological material generated in this study or from other sources.

The previously identified *fetC* and *ftrA* genes in *E. festucae* Fl1 form a gene cluster with an approximately 1.3‐kb intergenic region containing gene promoters for both genes (Johnson et al., 2013). The *fetC* locus is a 2.7‐kb stretch of genomic DNA with a coding sequence of 1.9 kb (Figure S1). BLAST analyses using the computed protein sequence of FetC from *E. festucae* Fl1 as a query against *S. cerevisiae* proteins or using the *S. cerevisiae* Fet3p protein (accession AJS70674.1) as a query against the proteins and genome of *E. festucae* Fl1 showed high sequence identity between them (49%). These results and the colocation with the *ftrA* gene suggest that *fetC* is the only gene encoding the multicopper ferroxidase involved in RIA in *E. festucae* Fl1.

### 2.2 Colony growth in response to iron supply

**FIGURE 1:**
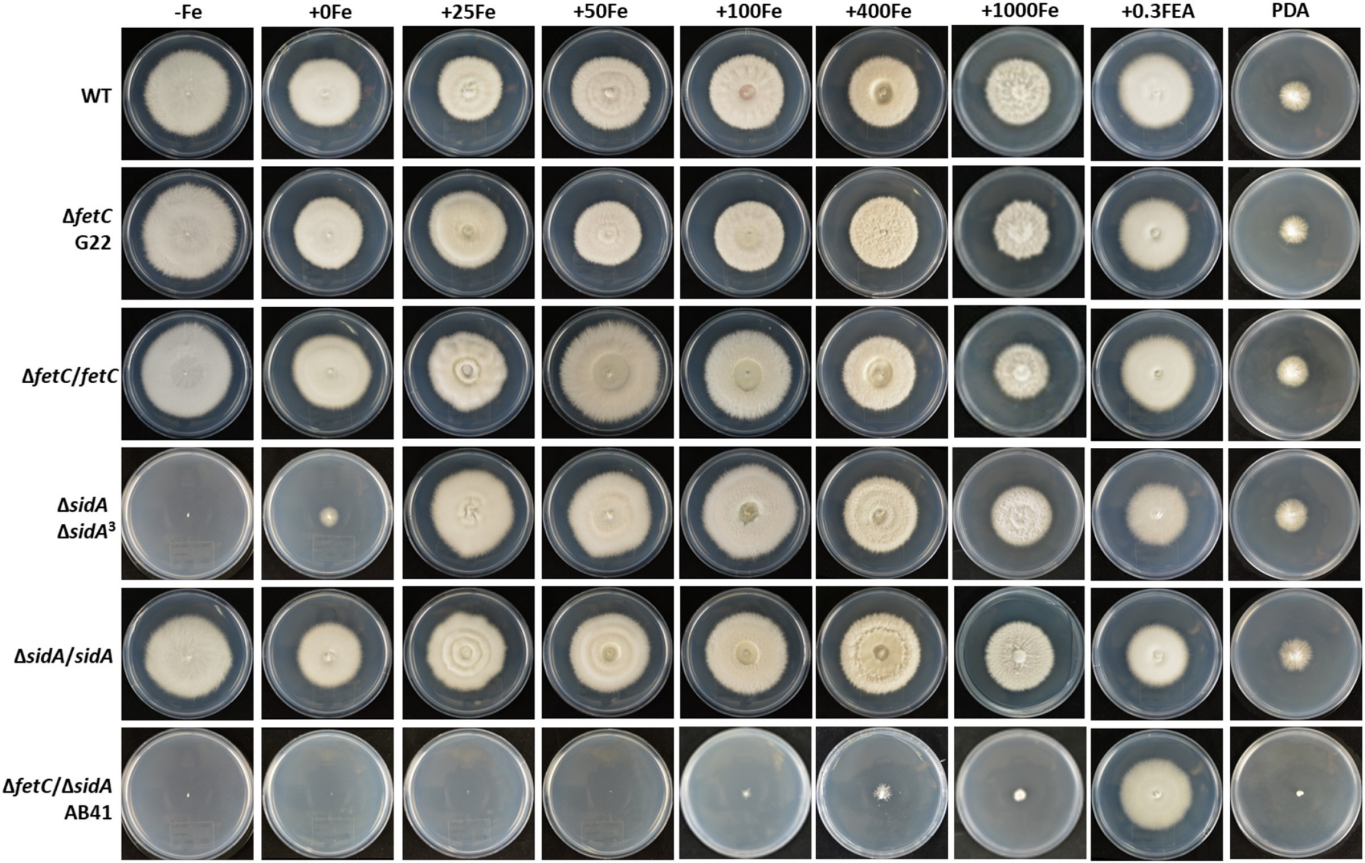
Growth morphologies of *Epichloë festucae* wild type (WT) and its derivatives. Strains WT, ∆ *fetC* , ∆ *fetC* / *fetC* , ∆ *sidA* , ∆ *sidA* / *sidA*, and ∆ *fetC* /∆ *sidA* were grown for 30 days at 22°C on defined medium; with iron chelation (−Fe: 100 μM bathophenanthroline disulphonic acid), with varied iron concentrations (+0Fe: 0 μM FeCl_3_ , +25Fe: 25 μM FeCl_3_ , +50Fe: 50 μM FeCl_3_ , +100Fe: 100 μM FeCl_3_ , +400Fe: 400 μM FeCl_3_ , +1000Fe: 1000 μM FeCl_3_ ), or supplemented with ferriepichloënin A (FEA, 0.3 μM). PDA, potato dextrose agar.

**FIGURE 2:**
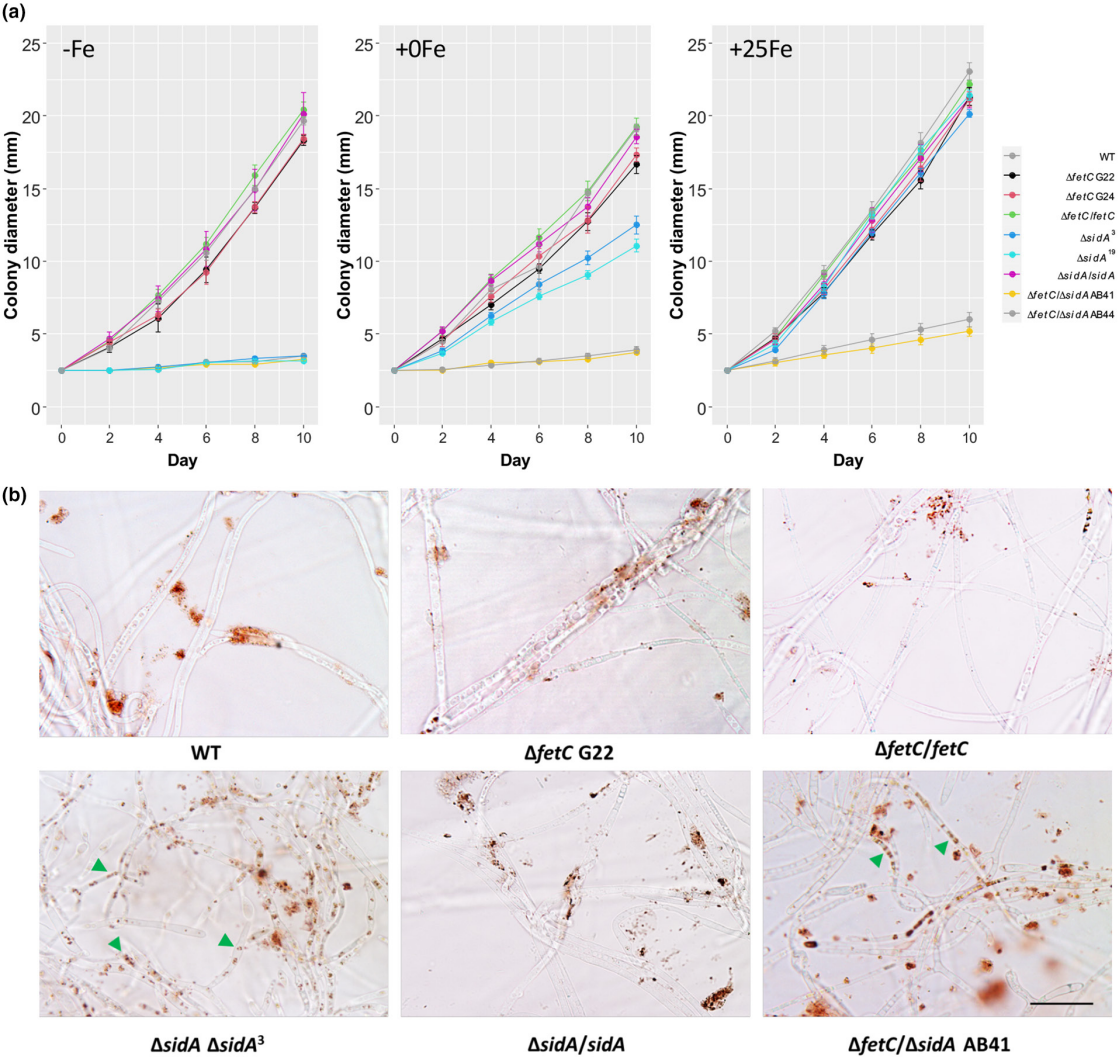
Colony growth rates and 3,3′‐diaminobenzidine (DAB) staining of H_2_O_2_ formed in hyphae of *Epichloë festucae* wild type (WT) and its derivatives. (a) Colony growth rates of *E. festucae* grown at 22°C for 10 days under defined medium; with iron chelation (−Fe: 100 μM bathophenanthroline disulphonic acid) or with varied iron concentrations (+0Fe: 0 μM FeCl_3_ , +25Fe: 25 μM FeCl_3_ ). Standard errors calculated based on six colonies per treatment are shown. (b) DAB staining of H_2_O_2_ formed in hyphae after 10 days of growth at 22°C on −Fe medium. Scale bars = 20 μm. Green arrowheads show DAB staining of H_2_O_2_ accumulated inside hyphae.

For all iron concentrations, the colony features of Δ*fetC* and the gene‐complemented strains, Δ*fetC*/*fetC* and Δ*sidA*/*sidA*, showed a similar morphology to that of the wild type (WT) over the study period (Figures 1 and 2). Analysis of colony growth indicated that Δ*fetC* colonies grew at a slightly slower rate than WT and gene‐complemented strains over the 10‐day measurement period. Beginning from Day 6, Δ*fetC* growth was significantly reduced (88%–89% of WT) until Day 10 on defined medium with iron chelation and from Day 8 (86%–90% of WT) on defined medium with 0 and 25 μM FeCl_3_, as determined using a least significant difference (LSD) test (*p* < 0.05) (Figure 2a). Consistent with a study by Forester et al. (2018), Δ*sidA* growth was most delayed under iron starvation, with radial and aerial hyphal growth becoming gradually restored with increasing iron concentration (>400 μM) (Figures 1 and 2).

The growth of Δ*fetC*/Δ*sidA* colonies was retarded in all growth conditions, even on potato dextrose agar (PDA). Iron starvation severely limited the growth of Δ*fetC*/Δ*sidA*, which barely grew after 30 days (Figure 1). A small increase in colony diameter was recorded with increasing iron concentrations at Day 10 (17%–18%, 19%–20%, and 22%–26% of WT under defined medium with iron chelation, 0 μM FeCl_3_, or 25 μM FeCl_3_, respectively; Figure 2), with all measurements being significantly different from WT, Δ*sidA*, Δ*fetC*, and their complemented strains. From Day 2 to Day 10, all strains showed significantly higher growth rates than Δ*fetC*/Δ*sidA* when the FeCl_3_ concentration was increased to 50 μM. However, there were no significant differences in the growth rates of WT, Δ*fetC*, Δ*sidA*, and the complemented strains under these conditions (Figure S2). Despite Δ*sidA* having a growth rate similar to that of WT, it exhibited a deficiency in aerial hyphae (Figure S3).

Like Δ*sidA*, the Δ*fetC*/Δ*sidA* strains increased colony growth rates with iron addition or FEA compared to iron‐depleted conditions, but colony diameters were still smaller than that of Δ*sidA* at any iron concentration (Figure 1). With the exogenous addition of FEA, Δ*fetC*/Δ*sidA* colonies restored radial but not aerial growth, as FC but not FEA is required for the formation of aerial hyphae (Forester et al., 2018). Furthermore, under defined medium with 1000 μM FeCl_3_, which is considered as a toxic level of iron for *Epichloë*, survival rates for the WT and gene‐complemented strains together averaged 31.9%, compared to 35.4%, 50%, and 12.5% for the Δ*fetC*, Δ*sidA*, and Δ*fetC*/Δ*sidA* strains, respectively.

Given the lack of aerial hyphae in Δ*sidA* and the extremely slow growth of Δ*fetC*/Δ*sidA* on PDA, hyphal morphology was examined. Microscopy revealed normal chitin accumulation at the hyphal tips and no differences were observed among all strains. The hyphae of the WT and the Δ*fetC* and Δ*sidA* mutants were rarely branched but frequently fused. Conversely, the hyphae of the Δ*fetC*/Δ*sidA* mutant were highly branched and sporadic swelling of compartments occurred with no hyphal fusion detected (Figure S4). Moreover, H_2_O_2_ accumulation was more frequently observed in Δ*fetC*/Δ*sidA* and Δ*sidA* hyphae than in WT and Δ*fetC* hyphae. Additionally, Δ*sidA* hyphae appeared to accumulate more H_2_O_2_ than Δ*fetC*/Δ*sidA* (Figure 2b), suggesting that Δ*sidA* is under greater oxidative stress than Δ*fetC*/Δ*sidA*.

### 2.3 Iron‐dependent siderophore production in *E. festucae*

As expected, no siderophore production was detected in any sample derived from Δ*sidA* or Δ*fetC*/Δ*sidA* strains (Figures S5 and S6). Two‐way analysis of variance (ANOVA) showed that there were significant interactive effects of *Epichloë* strain and growth medium on the production of intracellular FC (*p* < 0.05) and FEA (*p* < 0.05) siderophores. The greatest significant difference in the accumulation of FC between Δ*fetC* and WT as well as gene‐complemented strains was observed when iron supply was increased to 100 μM (*p* < 0.05). For the siderophore EA, under defined medium with bathophenanthroline disulphonic acid (BPS) and 100 μM FeCl_3_, the accumulation of intercellular total EA (as FEA) in Δ*fetC* mycelia was significantly higher compared to the other strains (*p* < 0.05). In terms of the culture supernatants, Δ*fetC* produced the greatest concentrations of the secreted EA under iron‐depleted conditions, while the other strains produced trace or very low concentrations of FEA under all iron concentrations (Table 1).

**TABLE 1:** Production of siderophores by *Epichloë festucae* in response to iron availability. Quantification of intracellular ferricrocin (FC), ferriepichloënin A (FEA) in mycelia, and extracellular FEA in culture supernatant extracts of *E. festucae* wild type (WT), Δ*fetC*, Δ*fetC*/*fetC*, and Δ*sidA*/*sidA* grown on defined medium with iron chelation (100 μM bathophenanthroline disulphonic acid [BPS]) or varied iron concentrations (0, 25, 50, and 100 μM FeCl_3_).

Added iron or iron chelator	Strain	Intracellular FC (pmol/mg)	Intracellular FEA (pmol/mg)	Extracellular FEA (μM)
100 μM BPS	∆*fetC* G22	8.1 ± 4.0 cd	101.9 ± 11.0 a	2.22 ± 1.12
	∆*fetC* G24	10.4 ± 6.2 cd	86.9 ± 13.8 a	2.15 ± 0.75
	WT	4.1 ± 2.3 cd	62.2 ± 14.8 b	1.03 ± 0.81
	∆*fetC*/*fetC*	7.4 ± 2.3 cd	53.0 ± 2.6 bc	Tr
	∆*sidA*/*sidA*	4.4 ± 1.4 cd	38.4 ± 7.4 cd	Tr
0 μM FeCl_3_	∆*fetC* G22	2.9 ± 1.7 d	16.0 ± 5.1 ef	Tr
	∆*fetC* G24	3.9 ± 2.8 d	24.9 ± 15.9 de	0.58 ± 0.26
	WT	6.9 ± 5.6 cd	5.8 ± 1.5 f	Tr
	∆*fetC*/*fetC*	7.4 ± 2.6 cd	5.4 ± 2.4 f	Tr
	∆*sidA*/*sidA*	6.0 ± 3.6 cd	9.3 ± 7.2 ef	Tr
25 μM FeCl_3_	∆*fetC* G22	6.3 ± 3.6 cd	19.5 ± 7.2 ef	Tr
	∆*fetC* G24	4.5 ± 1.4 cd	13.4 ± 4.0 ef	0.32 ± 0.25
	WT	3.4 ± 0.7 cd	1.2 ± 0.2 f	Tr
	∆*fetC*/*fetC*	6.6 ± 2.4 d	2.6 ± 0.6 f	Tr
	∆*sidA*/*sidA*	4.4 ± 1.3 cd	2.4 ± 1.1 f	Tr
50 μM FeCl_3_	∆*fetC* G22	11.2 ± 3.6 cd	16.7 ± 4.2 ef	Tr
	∆*fetC* G24	8.5 ± 2.0 cd	15.2 ± 3.2 ef	Tr
	WT	6.4 ± 3.7 cd	3.7 ± 2.5 f	Tr
	∆*fetC*/*fetC*	10.2 ± 1.5 cd	5.4 ± 2.3 f	Tr
	∆*sidA*/*sidA*	10.0 ± 3.1 cd	3.9 ± 1.8 f	Tr
100 μM FeCl_3_	∆*fetC* G22	30.9 ± 1.1 a	26.4 ± 2.1 de	Tr
	∆*fetC* G24	27.1 ± 5.4 b	26.9 ± 1.1 de	0.19 ± 0.08
	WT	12.0 ± 3.7 c	4.2 ± 1.2 f	Tr
	∆*fetC*/*fetC*	9.5 ± 3.8 cd	6.2 ± 3.2 f	Tr
	∆*sidA*/*sidA*	11.7 ± 4.9 cd	3.9 ± 1.5 f	Tr

*Note*: Different letters for each siderophore indicate a statistical difference between treatments based on the LSD test. The limit of detection (LOD) and the limit of quantification (LOQ) were 0.6 and 2 pmol/mg for both intracellular FC and FEA and 0.05 and 0.15 μM for extracellular FEA, respectively. Tr denotes trace levels (mean < LOQ). For all samples, extracellular FC was not detected.

### 2.4 Hyphal growth characteristics in the host plant

**FIGURE 3:**
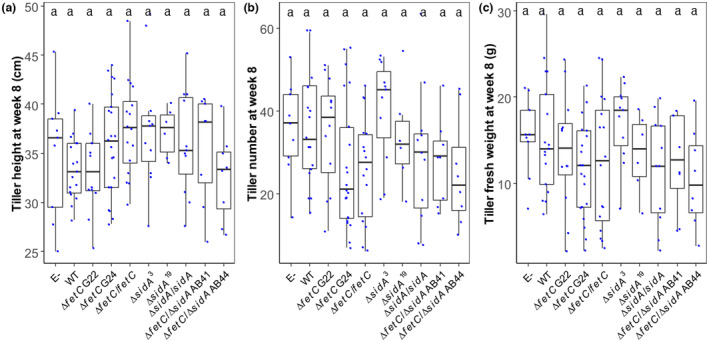
Boxplots of (a) tiller height, (b) tiller number, and (c) plant fresh weight of ryegrass plants 8 weeks after planting. Plants were mock‐inoculated (E−) or infected with *Epichloë festucae* wild type (WT), Δ*fetC* , Δ*fetC* / *fetC* , Δ*sidA* , Δ*sidA* / *sidA*, and Δ*fetC* /Δ*sidA* . Each blue dot represents one biological replicate. Different letters on the bars indicate significant differences between strains as determined using the LSD test (*p* < 0.05).

**FIGURE 4:**
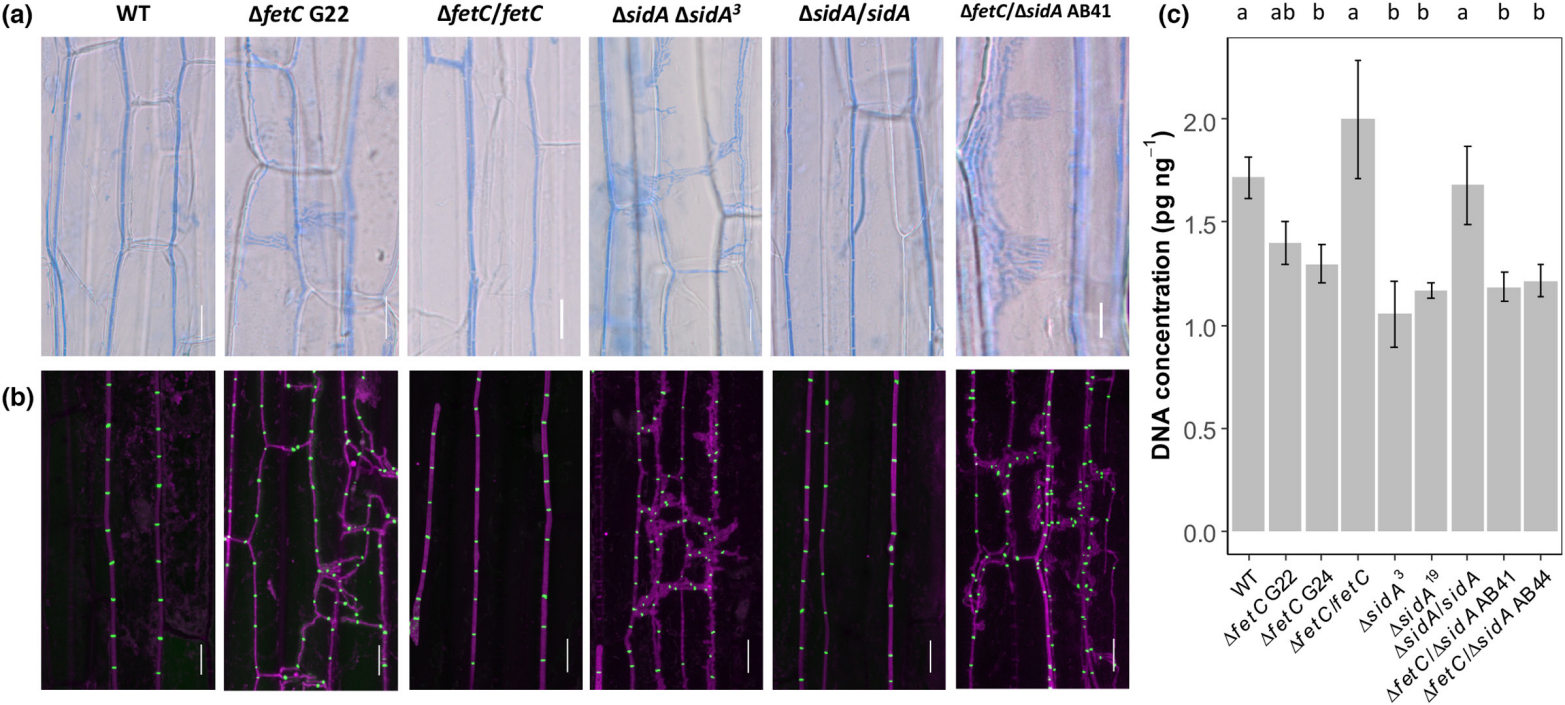
Hyphal growth in the leaf sheath and endophyte biomass in the pseudostem tissues. Leaf sheaths were taken from ryegrass plants infected with *Epichloë festucae* wild type (WT), ∆ *fetC* , ∆ *fetC* / *fetC* , ∆ *sidA* , ∆ *sidA* / *sidA*, and ∆ *fetC* /∆ *sidA* and analysed by (a) light microscopy and (b) confocal laser scanning microscopy. Confocal microscopy images of aniline blue‐stained hyphae (purple pseudocolour) and septa labelled with WGA‐488 (green pseudocolour). Bars = 20 μm. (c) DNA concentrations (pg endophyte DNA per ng total [plant + endophyte] DNA) in the basal pseudostem tissues of ryegrass plants. Different letters beside the bars indicate significant differences between treatments as determined using the LSD test (*p* < 0.05).

Neither infection with *E. festucae* WT nor the disruption of *fetC*, *sidA* or *fetC*/*sidA* in *E. festucae* had a significant effect on perennial ryegrass growth parameters including tiller height (*p* = 0.107 by ANOVA), tiller number (*p* = 0.081 by ANOVA), and plant fresh weight at Week 8 (*p* = 0.263 by ANOVA) (Figure 3). However, sporadically occurring structures comprising hyperbranched hyphae were observed in leaf sheath tissues that were colonized by Δ*fetC*, Δ*sidA*, and Δ*fetC*/Δ*sidA* mutants under both light and confocal microscopy (Figure 4a,b). In comparison, the hyphae of the WT and gene‐complemented strains grew parallel to the leaf axis and were rarely branched, which is consistent with normal growth (Christensen et al., 2002). The ratio of *E. festucae* DNA to total DNA was significantly reduced in pseudostem tissues infected with Δ*fetC* (1.3 pg/ng), Δ*sidA* (1.1 pg/ng), and Δ*fetC*/Δ*sidA* (1.2 pg/ng) compared to WT (1.7 pg/ng) (*p* < 0.05 using Dunn's multiple comparison test) (Figure 4c).

**FIGURE 5:**
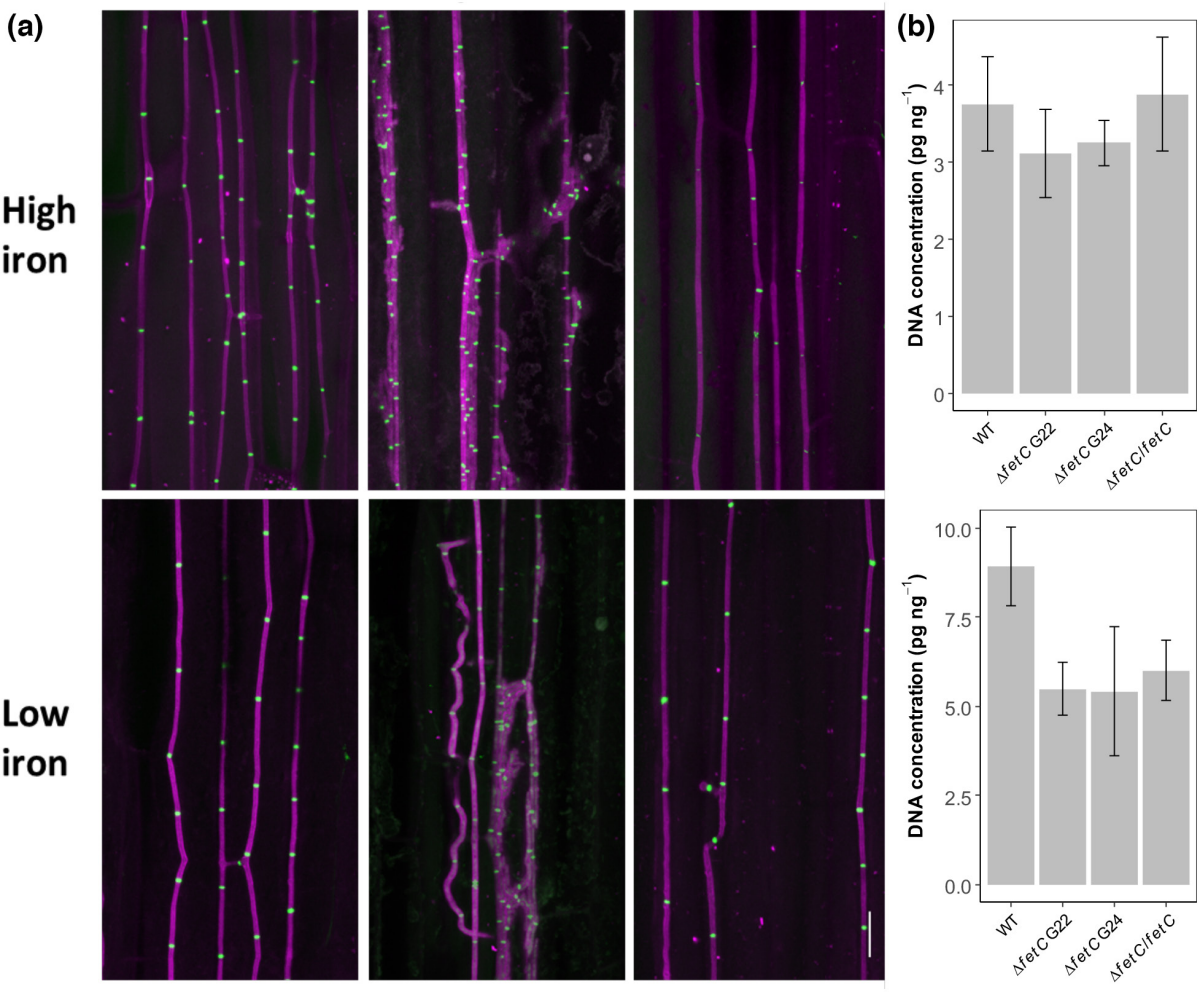
Hyphal growth of *Epichloë festucae* in ryegrass plants infected with wild type (WT), ∆ *fetC*, and ∆ *fetC* / *fetC* grown in high‐ and low‐iron conditions. (a) Confocal microscopy images of aniline blue‐stained hyphae (purple pseudocolour) and septa labelled with WGA‐488 (green pseudocolour). Bars = 20 μm. (b) Endophyte DNA concentration (pg/ng total DNA) in the pseudostem tissues of plants infected with WT, ∆ *fetC*, and ∆ *fetC* / *fetC* .

To understand the effect of iron supply on the growth of Δ*fetC* within ryegrass tissues, a hydroponic experiment was conducted comparing high‐iron (50 μM) versus low‐iron (0.5 μM) treatments. No changes in host phenotypes were observed between plants infected with WT or Δ*fetC* under either high‐ or low‐iron conditions (data not shown). Similar to soil‐grown plants, endophytic hyphae from WT and gene‐complemented strains were straight and resided in the intercellular spaces of the leaf sheath, whereas those of Δ*fetC* often displayed highly branched hyphae under both high‐ and low‐iron conditions (Figure 5a). DNA quantification of *E. festucae* showed that differences in hyphal biomass in pseudostem tissues infected with WT, Δ*fetC* G22, Δ*fetC* G24, or Δ*fetC*/*fetC* under high‐iron conditions were nonsignificant (*p* = 0.76). However, under low‐iron concentrations, the in planta hyphal biomass in both Δ*fetC* strains‐infected pseudostem tissues (5.5 pg/ng total DNA) was lower than in WT‐infected pseudostem tissues (8.9 pg/ng total DNA), but there was no statistical difference between the groups (*p* = 0.09) (Figure 5b).

### 2.5 Disruption of iron assimilation genes reduced host seed yield and *Epichloë* transmission

The average reproductive tiller percentage was significantly affected by the disruption of iron assimilation systems (*p* = 0.01); it was significantly lower in perennial ryegrass infected with mutant strains (Δ*fetC*, 14%; Δ*sidA*, 11%; Δ*fetC*/Δ*sidA*, 23%) than in perennial ryegrass infected with WT (41%) or mock inoculated (36%), as determined using Dunn's multiple comparison test (Figure S7a). However, the disruption of genes involved in either siderophore or RIA‐mediated iron uptake had no effect on the spike length (*p* = 0.34), the total spikelet number per spike (*p* = 0.45), the fertile spikelet number per spike (*p* = 0.52), or the fertile spikelet percentage (*p* = 0.18) (Figure S7b–e). Furthermore, no significant differences in any of the flowering features measured were observed between ryegrass plants with mock inoculation and infected with WT using Dunn's multiple comparison test.

**FIGURE 6:**
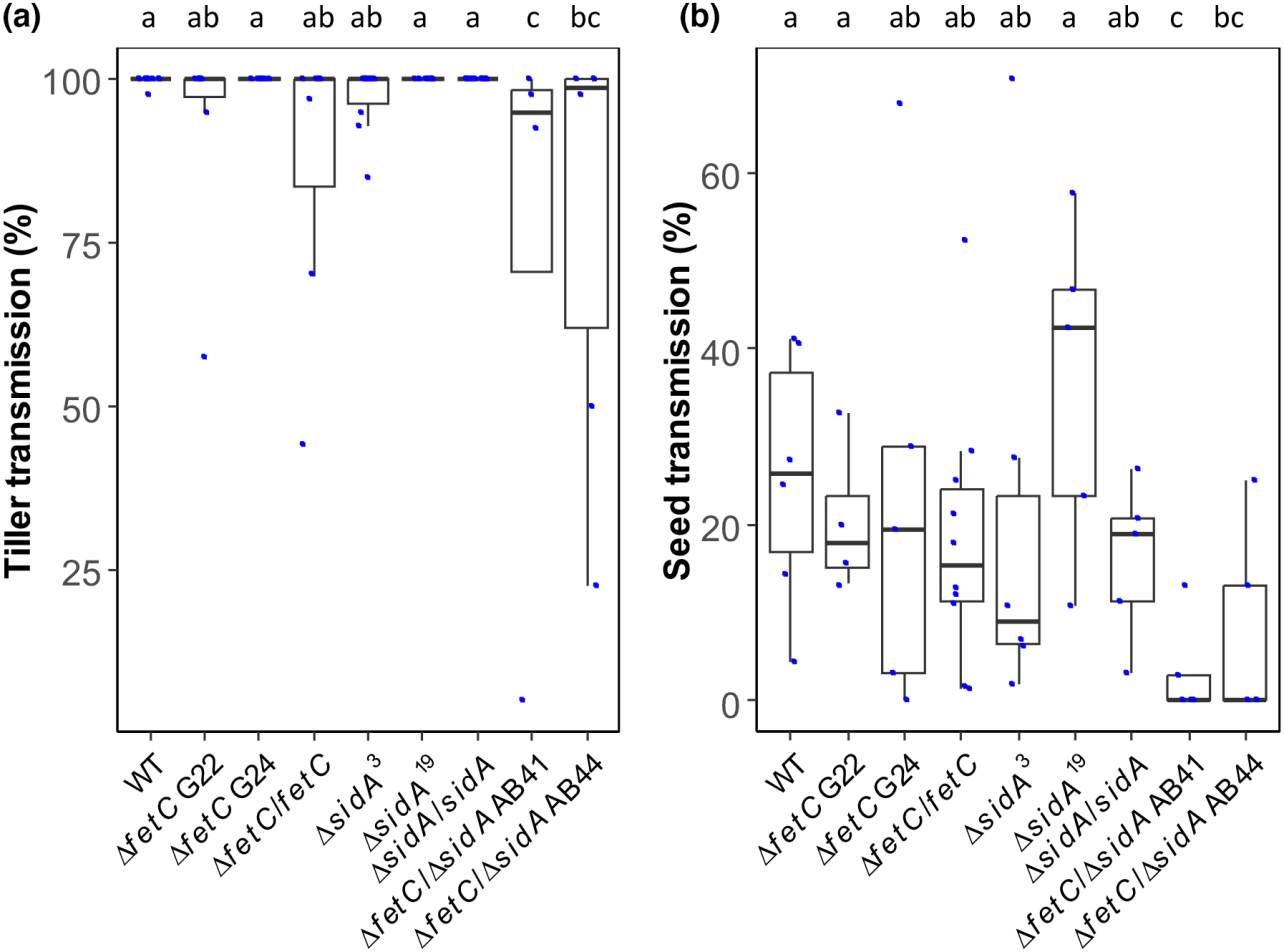
Endophyte tiller and seed transmission in ryegrass plants infected with *Epichloë festucae* wild type (WT) and its derivatives. Boxplots illustrating (a) relative tiller infection levels in parent plants and (b) relative viable endophyte transmission into seedlings after infection with WT, ∆ *fetC* , ∆ *fetC* / *fetC* , ∆ *sidA* , ∆ *sidA* / *sidA*, and ∆ *fetC* /∆ *sidA* . Each blue dot represents one biological replicate. The different letters on the bars indicate significant differences between strains as determined using the LSD test (*p* < 0.05).

Endophyte transmission to daughter tillers was reduced in plants infected with the Δ*fetC*/Δ*sidA* mutant (AB41: 73%; AB44: 78%) compared to that in plants infected with other strains (WT, 99%; Δ*fetC* [G22: 93%, G24: 100%]; Δ*fetC*/*fetC*, 87%; Δ*sidA* [Δ*sidA* ^*3*^: 97%, Δ*sidA* ^*3*^: 100%]; Δ*sidA*/*sidA*, 100%). As expected, daughter tillers and seeds harvested from mock‐inoculated ryegrass plants continued to be free of *Epichloë* infection (0%), as confirmed using the tissue‐print immunoblotting and seed squash methods, respectively (data not shown). For WT, the seed transmission rate (25%) was much lower than the vegetative infection rate (99%), but it was significantly higher than that of Δ*fetC*/Δ*sidA*‐infected seeds (AB41: 3%; AB44: 8%) (*p* < 0.05) as determined using Dunn's multiple comparison test (Figure 6a,b). The results indicate that maintenance of endophyte iron metabolism is important for the reproductive tiller percentage and vertical endophyte transmission.

## 3 DISCUSSION

Iron is a micronutrient that is required for the mutualistic symbiosis between perennial ryegrass and *E. festucae*. Previous research has established that the *E. festucae* siderophore system, which consists of the cellular siderophore FC and the dual localized siderophore EA, is essential for *E. festucae* survival in culture and also for the mutualistic interaction (Forester et al., 2018; Johnson et al., 2013). Through abolishing RIA in *E. festucae* by deletion of *fetC*, in culture developmental delays were observed under iron‐depleted conditions, which were exacerbated when in combination with the Δ*sidA* mutation (i.e., Δ*fetC*/Δ*sidA*), thus establishing a role for FetC in iron‐dependent growth. Growth retardation of Δ*fetC* was less severe than that of Δ*sidA* and Δ*fetC*/Δ*sidA* when iron was omitted (Figure 2). Because both high‐affinity iron uptake systems are active under low‐iron conditions, we inferred the different contributions of RIA and siderophore‐mediated iron metabolism through the phenotypic differences observed. For example, the sparse growth of Δ*sidA* on defined medium is probably supported by RIA in the absence of siderophores, whereas siderophores appear to support the growth of Δ*fetC*. We have demonstrated that extracellular siderophores are responsible for the recovery of growth in Δ*fetC*/Δ*sidA* mutants supplemented with FEA, similar to the results of previous siderophore complementation tests (Forester et al., 2018). Furthermore, exogenous iron supplementation (>100 μM) fully restored the colony phenotypes of Δ*sidA* and partially restored those of Δ*fetC*/Δ*sidA* (Figure 1), which suggests the presence of alternative low‐affinity iron uptake systems, like those seen in certain other fungi. *S. cerevisiae* for example uses the Fe(II) permease (Fet4p) protein (Hassett et al., 2000), whereas the rice false smut pathogen *Ustilaginoidea virens* uses Uvt3277 as the low‐affinity iron transporter (Zheng et al., 2017), and an as yet to be characterized low‐affinity iron uptake system has also been proposed for *A. fumigatus*, which are also viable in the absence of high‐affinity uptake systems (Schrettl et al., 2004). Therefore, we conclude that siderophore‐mediated iron uptake is the dominant strategy in *E. festucae*, at least in culture, and that low‐affinity uptake systems are likely to exist.

*E. festucae* produces two siderophores, FC and EA (Johnson et al., 2013). Our previous research showed that FC is the primary intracellular siderophore under iron‐sufficient conditions, while EA is the dominant intracellular and secreted (extracellular) siderophore when iron access is limited (Forester et al., 2018; Johnson et al., 2013). Concomitant with those studies, our results (Table 1) suggest that siderophore‐assisted iron assimilation is greater in Δ*fetC* mutants than in WT. Under low‐iron conditions, the significantly higher EA concentrations suggest that Δ*fetC* is more iron‐starved than WT, but the higher concentrations of FC suggest less restricted access to iron by FC. Conversely, under iron‐sufficient conditions, the sustained high concentrations of intracellular EA and accumulation of FC suggest an increased requirement for iron sequestration by FC, as the iron internalized as FEA can be transferred to FC, thus allowing EA to be recycled. Therefore, it could be that the mechanisms that coordinate siderophore production and transport are partly deregulated by the lack of RIA activity.

In the absence of extracellular siderophores, EA enhances the formation of H_2_O_2_ in the iron‐depleted Δ*sidA* and Δ*sidN* mutants (Forester et al., 2018; Johnson et al., 2013). Further, this study has found little effect of deletion of *fetC* alone, but the Δ*fetC*/Δ*sidA* mutants appeared to form less H_2_O_2_ precipitates compared to Δ*sidA*, which implies that the RIA system adds to oxidative stress under low‐iron conditions (Figure 2b). Release of free iron by RIA into the cell without interception by siderophores may be the reason, with the Δ*fetC* mutants ameliorating some iron stress through the absence of the RIA system in combinatorial mutants and also via enhanced production of siderophores, particularly EA in the single Δ*fetC* mutants (Table 1). This further supports the protective role of EA against reactive oxygen stress in *E. festucae*. Similar to our results, in studies of *C. heterostrophus*, the combinatorial deletion of the RIA component FTR1 and the extracellular siderophore nonribosomal peptide synthetase enzyme NPS6 (but not the intracellular siderophore biosynthetic enzyme NPS2) led to hypersensitivity to oxidative stress, but at the same levels as the *nps6* mutant alone (Condon et al., 2014). To minimize free excess iron stimulation of hydroxyl radical formation (Fenton reaction) (Halliwell et al., 1992; Schützendübel & Polle, 2002), the balanced interplay between the siderophore and RIA systems in *E. festucae* is probably important to maintain a metabolic balance between iron uptake and iron sequestration.

All mutant strains lacking one or two high‐affinity iron uptake systems displayed aberrant hyphal morphologies in leaf sheath tissues of infected host plants and reduced biomass (Figures 4 and 5). The reduced *Epichloë* biomass of all mutant‐infected ryegrass plants was associated with swollen, branched hyphae and epiphyllous hyphal structures in the pseudostem tissues. It appeared from the microscopic images that the biomass of *E. festucae* mutant strains may be higher than that of the WT, which conflicted with the DNA quantification results. This phenomenon could suggest a patchy growth pattern of the mutant strains that lack one type of high‐affinity iron uptake system. These strains could colonize more in iron‐enriched regions (e.g., xylem vessels) (Ariga et al., 2014) as areas with low iron concentrations probably do not support mutant hyphal growth. We also found that for both WT and Δ*fetC*, endophyte biomass was higher under low‐iron than under high‐iron conditions, which might be due to a dilution effect, presuming an increase of grass growth and no change in fungal growth under high‐iron conditions. Similar results were found in ryegrass plants under low nitrogen and phosphate supply (Liu et al., 2011; Rasmussen et al., 2007).

Hyphal biomass has been shown to be correlated with seed transmission (Albarouki et al., 2014; Gagic et al., 2018), and we hypothesized that, because Δ*fetC*, Δ*sidA*, and Δ*fetC*/Δ*sidA* had reduced biomass, they would not colonize new tillers or transmit to the seed as well as the WT strain. However, this was true only for Δ*fetC*/Δ*sidA*, in which vegetative and floral transmission was significantly reduced relative to WT (Figure 6), suggesting that possessing functional RIA (in Δ*sidA*) or siderophore (in Δ*fetC*) systems can support *Epichloë* transmission. The failure to form hyphal fusions in Δ*fetC*/Δ*sidA* (Figure S4) might be indicative of a restrictive growth pattern of *E. festucae* in symbiosis with ryegrass plants, suggesting that host colonization could be defective (Becker et al., 2014). This further demonstrates the critical role of iron for balancing host–endophyte compatibility and that both high‐affinity iron uptake systems are required for normal in planta growth. Although we saw a decrease in *Epichloë* transmission in Δ*fetC*/Δ*sidA*‐infected ryegrass plants, the tillers and seed progeny derived from Δ*fetC*/Δ*sidA* did not become entirely endophyte‐free. Therefore, we surmise that the presence of low‐affinity iron uptake systems in *E. festucae* may be able to partially sustain systemic Δ*fetC*/Δ*sidA* infection in plants. Another hypothesis is that *E. festucae* might utilize siderophores secreted by other microorganisms living inside or outside of the host plants to assimilate iron. There is evidence showing that some fungal species, for example, *Candida albicans*, *Aspergillus nidulans*, and *S. cerevisiae*, can utilize xenosiderophores, siderophores produced by other microorganisms (Haas, 2003; Heymann et al., 2002; Philpott & Protchenko, 2008).

In contrast to some pathogens that appear to prioritize the use of one type of iron uptake mechanism over another (Burbank et al., 2015; Eichhorn et al., 2006), this study confirms that *E. festucae* uses both high‐affinity iron uptake systems concurrently in iron metabolism, with some level of redundancy to maintain iron homeostasis, similar to *C. graminicola* and *C. heterostrophus* (Albarouki et al., 2014; Albarouki & Deising, 2013; Condon et al., 2014). This redundancy confers *E. festucae* more flexibility, with increased capacity to obtain and use iron from its environment. Similar findings have been reported for *C. heterostrophus*, a maize pathogen, where the role of RIA in pathogen virulence is overshadowed by the involvement of extracellular siderophores as a high‐affinity iron uptake mechanism, with RIA serving as a critical backup for the fungus (Condon et al., 2014). We found in this study that disruption of either one or two high‐affinity iron uptake systems reduced the percentage of reproductive tillers in flowering plants. This may reflect reduced floral induction and/or termination of inflorescence development through somehow impaired interactions with these mutants. Moreover, the complemented strains for ∆*fetC* and ∆*sidA* did not fully recover the phenotype as observed in the WT‐infected plants (Figure S7). The likely reason for this was because the complementation method was performed through ectopic integration of the gene into a non‐native genomic location, which may cause the overall function of the complemented gene to differ from its native context, particularly for *sidA* and *fetC*, which both form part of a divergently coexpressed gene pair; the physical decoupling of the genes may prevent full complementation. Therefore, the differences in reproductive tiller percentage between plants infected with WT or treated with mock inoculation and the complemented strains are reasonable. Additionally, the design of p∆*fetC* involved the deletion of an approximately 500‐bp intergenic region including the *fetC* promoter sequences (Figure S1), which may have also impacted the gene expression of *ftrA* in ∆*fetC* and thereafter ∆*fetC*/*fetC* under iron‐depleted conditions. The gene expression of *ftrA* (Figure S8) appeared reduced, but the increased FC concentration in these strains under iron deficiency (Table 1) might also suggest that higher iron availability could lead to reduced expression of the iron deprivation‐responsive *ftrA* gene (Schrettl et al., 2008). The disruption of either RIA or siderophore systems not only perturbs endophyte hyphal phenotype and biomass levels in the host interaction, but also impedes *Epichloë* transmission between tillers and to the subsequent seed generation, which impacts fitness. Seed transmission of ∆*fetC*/∆*sidA* may have been affected by the decrease in host tiller infection. Future transcriptomic analyses may identify the molecular factors and processes involved in the loss of *Epichloë* endophyte transmission when both iron uptake systems are abolished. Moreover, it would be interesting to explore the role of siderophores and RIA in seed transmission of other strains of *Epichloë* that have high levels of transmission (that of the Fl1 WT strain was only 25%) (Figure 6), such as the commercially available *Epichloë* strains that are required to be sold with an endophyte viability level above 70% (Caradus & Johnson, 2020). For *E. festucae*, both siderophores and RIA are functional iron assimilation systems with fundamental roles in iron homeostasis for growth, which in turn preserves the symbiotic interactions during host grass vegetative and reproductive development.

In this study, we have examined the role of the high‐affinity iron uptake system RIA as a complementary iron uptake system in *E. festucae* Fl1. Although some redundancy with the siderophore iron uptake system is apparent, the RIA system can also contribute independently to endophyte iron uptake under both in culture and in planta growth conditions. However, the loss of both high‐affinity uptake systems critically affects the *Epichloë* ryegrass interaction phenotype and the transmission of *Epichloë* during the plant life cycle.

## 4 EXPERIMENTAL PROCEDURES

### 4.1 Plant and *Epichloë* growth conditions and endophyte inoculations

*E. festucae* Fl1 (wild‐type [WT] strain) was originally isolated from perennial ryegrass cultivar SR3000 (Leuchtmann et al., 1994, 2014). All *E. festucae* strains were maintained on 3.9% (wt/vol) PDA (Oxoid Ltd) at 22°C in an 8 h light/16 h dark cycle. Defined medium (Johnson et al., 2013) was used as a nutritional base for fungal growth assays and chemical analyses. Endophyte‐free seeds for *Epichloë* inoculation were sourced from the Margot Forde Germplasm Centre (Palmerston North, New Zealand). Fungal saprophytes and other seedborne endophytes were removed by heat treatment using the method of Bouton et al. (1993) with extended incubation (47°C for 3 weeks at 45% relative humidity). Seed packets were placed in a desiccator jar on a gauze mat above 100 mL of a 75% glycerol and 25% water mixture (vol/vol) to maintain relative humidity. *Epichloë* inoculation into perennial ryegrass seedlings was previously described (Becker et al., 2018; Latch & Christensen, 1985). Tissue‐print immunoblotting was used to confirm *Epichloë* infection (Simpson et al., 2012). *Epichloë*‐infected perennial ryegrass plants were grown in 1‐L pots filled with potting medium, kept in a glasshouse, and watered as required. Mature plants (12 weeks postinoculation, c.100 tillers/pot) were vernalized at 6°C for 10 weeks with a short‐day photoperiod (8 h light and 16 h dark). The plants were returned to ambient warm, long‐day conditions in the glasshouse, where they developed inflorescences 4–10 weeks later.

### 4.2 Construction, validation, and selection of deletion mutants

Details can be found in File S1.

### 4.3 In culture growth assay

All strains were evaluated for their ability to grow under a range of iron concentrations and culture conditions, in addition to complete medium (i.e., PDA). Defined medium (<1 μM iron with 2% agar, +0 Fe) was supplemented with BPS (Sigma‐Aldrich) to generate iron‐starvation medium (defined medium with 100 μM BPS [= −Fe]), or FeCl_3_ was added to generate adequate (25 μM), sufficient (50 and 100 μM), or excess (400 and 1000 μM) concentrations. To determine the influence of siderophores on colony development, 0.3 μM FEA was added to the defined medium. To assess the toxicity of iron to *Epichloë*, the survival rates (%) of all strains were determined by observing their growth on defined medium supplemented with 1000 μM FeCl_3_ using 24 plugs (150 mm length, tip diameter 1 mm, agar come with the inoculum) transferred from PDA (or PDA supplemented with 0.3 μM FEA for Δ*fetC*/Δ*sidA*) that were allowed to revive after 14 days.

To assess general colony growth characteristics on these media, colonies of all strains (except for Δ*fetC*/Δ*sidA*) were subcultured from PDA cultures using glass Pasteur pipettes (150 mm length, tip diameter c.1 mm, agar come with the inoculum) and incubated at 22°C for 30 days on defined medium with different iron concentrations or for 10 days on PDA, before being photographed. The Δ*fetC*/Δ*sidA* mutant was subcultured from PDA supplemented with 0.3 μM FEA because it grew extremely slowly on PDA.

Colony growth rate assays were performed on defined medium under −Fe conditions and on defined medium supplemented with 0, 25, 50, and 100 μM FeCl_3_. Colonies were transferred from PDA culture to specific defined medium using plastic transfer pipettes (150 mm length, tip diameter 2.5 mm, agar come with the inoculum) and incubated at 22°C for 10 days. Culture diameter was recorded every 2 days. An exception was made for Δ*fetC*/Δ*sidA*, which was subcultured from a PDA culture supplemented with 0.3 μM FEA. For each strain, six replicates were included (two plates containing three colonies each).

### 4.4 Measurements of siderophore concentrations

Starter cultures were grown in 100‐mL flasks containing 50 mL liquid defined medium for 10 days at 22°C, with shaking at 100 rpm. A 2‐mL aliquot of each culture was transferred to a 2‐mL microcentrifuge tube and centrifuged at 3000 *g* for 10 min. The supernatant was discarded, and the mycelial pellet was finely ground with a sterile micropestle (Sigma‐Aldrich) and inoculated into 50 mL fresh defined medium with sterile filtrates of BPS (100 μM) or FeCl_3_ (0, 25, 50, and 100 μM) within 100‐mL flasks. Cultures were grown for 5 days, transferred to 50‐mL centrifuge tubes, and centrifuged at 3000 *g* for 10 min. Mycelium was transferred to a 2‐mL screw cap microcentrifuge tube and centrifuged at 14,000 *g* for 20 min. The supernatant and mycelium were analysed separately for siderophores. A 100‐μL aliquot of supernatant (filtered through a 0.2‐μm membrane) was extracted with 200 μL siderophore extraction solvent A (75% methanol [vol/vol] with 1.5 mM FeCl_3_). Mycelium was freeze‐dried and extracted with siderophore extraction solvent B (50% methanol [vol/vol] with 1.5 mM FeCl_3_) at 50 mg/mL, followed by cell disruption at a speed of 4 m/s for a single cycle of 30 s in an Omni Bead Ruptor homogenizer (OMNI International). The supernatant was analysed after centrifugation at 14,000 *g* for 20 min. The method described by Forester et al. (2018) was used for the preparation of siderophore standards and the analysis of siderophores by liquid chromatography–mass spectrometry (LC‐MS). The solvent methanol used in this work for siderophore extraction and analysis on an LC‐MS platform was of HPLC grade. Standards of FC and desferricrocin were purchased from EMC Microcollections (Tübingen, Germany).

### 4.5 Microscopy of hyphae in culture and in planta

Using cultures grown on PDA for 7 days, we examined the morphology of hyphal tips and hyphal fusion. Hyphae were stained following the method described by Shoji et al. (2015). Images were taken with an FV1000‐D confocal laser scanning microscope (Olympus) and FV10i‐ASW 3.1 Viewer software (Olympus).

The accumulation of H_2_O_2_ within hyphae was investigated using a 10‐day‐old culture grown on defined medium containing 100 μM BPS. The accumulation of H_2_O_2_ was visualized as reddish‐brown stain formed following 3,3′‐diaminobenzidine (DAB) reaction with H_2_O_2_ (Thordal‐Christensen et al., 1997). Hyphae were stained with DAB and hyphae and plants were microscopically examined following the method described by Forester et al. (2018).

### 4.6 Quantification of *Epichloë* biomass in perennial ryegrass

The *Epichloë* biomass in pseudostem tissues was quantified by using a quantitative PCR (qPCR) method (Cook et al., 2009). *Epichloë* biomass is expressed as picograms of *Epichloë* DNA per nanogram of total (plant and *Epichloë*) DNA (i.e., pg/ng total DNA). The quantification of *Epichloë* DNA is based on a 153‐bp DNA fragment amplified from the nonribosomal peptide synthetase gene *NRPS1* (Rasmussen et al., 2007). For the quantification of the fungal endophyte, a standard curve (20, 10, 1, 0.1, 0.01, and 0.001 ng fungal DNA) was prepared from DNA extracted from a pure culture of the Fl1 WT strain extracted using the ZR Fungal/Bacterial DNA MiniPrep kit (Zymo Research Corp.). Three pseudostem tissues (1 cm above roots) from a single plant were pooled and at least four biological replicates were assayed on the plants infected with each construct. Total DNA was extracted from the pseudostem tissues using the NucleoSpin Plant II genomic DNA extraction kit (Macherey‐Nagel GmbH) and quantified using a Qubit fluorometer with the Quant‐iT dsDNA HS Assay kit (Invitrogen). qPCR was performed using KAPA SYBR FAST qPCR reagents (KAPA Biosystems) in a 10‐μL reaction containing total DNA (10 to 100 ng) and 200 nM each of the primers NRPS1_ qPCR_F and NRPS1_ qPCR_R in a Light‐Cycler 480 (Roche Applied Science).

A list of primers used in this study can be found in Table S2.

### 4.7 Gene expression quantification

Details can be found in File S1.

### 4.8 Flowering spike morphology and *Epichloë* seed transmission

At the seed ripening stage, the flowering spike length (cm), total and fertile spikelet numbers, seed weight per spike (g), and grain number per spikelet were determined. Spike length was determined by measuring the distance between the base of the most basal floret and the tip of the most apical floret. Fertile spikelets produce at least one grain, whereas infertile spikelets do not set any grain (Guo et al., 2017). Fertility is defined as the ratio of the fertile to total spikelet counts. For trait measurements, at least four plants infected with each transformant were randomly selected.

### 4.9 Measurement of vegetative and vertical transmission of *Epichloë*

To determine the efficiency of *Epichloë* transmission in vegetative tillers, 1‐year‐old plants infected with each strain were analysed for endophyte presence in the tillers by tissue‐print immunoassay. Approximately 40 randomly selected tillers per plant and at least eight plants infected with each construct were tested.

Seeds harvested from mock‐inoculated endophyte‐free ryegrass plants were analysed for the presence of *Epichloë* using a common seed squash method (Clark et al., 1983). To determine the rate of viable *Epichloë* transmission from maternal plants to seedlings, approximately 108 seeds from each maternal plant were sown and grown for 5 weeks until one to three tillers were present. Seedlings were then tissue‐print immunoblotted to assess infection status. At least five maternal parent plants infected with each construct were used, and the percentage of progeny seeds with viable endophyte was determined.

### 4.10 Growth assays of hydroponically grown plants

A hydroponic system was used to control nutrient availability and evaluate the impact of iron deficiency on plants infected with ∆*fetC* more precisely. Iron‐free hydroponic solution was prepared (Forester et al., 2018). The iron source was a chelated EDTA ferric sodium salt solution, which was used to create two distinct treatments (high iron: 50 μM Fe^3+^, low iron: 500 nM Fe^3+^) (Figure S9). Root‐trimmed mature tillers infected with WT, ∆*fetC*, and ∆*fetC*/*fetC* were transferred from soil to hydroponic conditions. Two independent 24‐site hydroponic propagation systems were set up with separate containers containing approximately 3.5 L hydroponic solution with two iron concentrations (Bothe et al., 2018). Three tillers of a single genotype were inserted into a neoprene plug at each site. For each inoculated treatment, four biological replicates and two technical replicates were included, which were randomly arranged in the propagators. To regenerate new roots, the tillers were grown for 10 days in hydroponic solution containing 20 μM iron, followed by 4 weeks in high‐ or low‐iron conditions. Hydroponic solutions were refreshed every 5 days.

### 4.11 Data analysis

RStudio (v. 1.1.447) and R software (v. 4.0.1) were used to conduct all statistical analyses (R Core Team, 2021; RStudio Team, 2020). Two‐way or one‐way ANOVA was conducted using the Anova function in the “car” package (Fox et al., 2007). An LSD method was adopted for further post hoc analysis with the “agricolae” package (de Mendiburu & de Mendiburu, 2019). Pairwise treatment comparisons were carried out with Fisher's protected LSD test (α = 0.05). Data that did not satisfy the assumptions of normality and homogeneity of variance were analysed by one‐way ANOVA with the nonparametric Kruskal–Wallis method using the “stats” package and Dunn's multiple comparison test with the “dunn.test” package. The data were plotted using either R functions or with the “ggplot2” package (Wickham, 2011).

## Supporting information


**FIGURE S1.** Constructs of *Epichloë festucae* Δ*fetC* and Δ*fetC*/Δ*sidA* strains. (a) Gene replacement of *fetC* with the *hph* cassette by homologous recombination. Long coloured arrows represent the transcript for each gene model EfM3.036730‐50 and short red arrows denote potential SreA transcription factor binding sites. (b) Replacement of the *sidA* gene with the *nptII* gene by homologous recombination. (c) PCR screening for the ∆*fetC* mutants with two primers (LJNTF3‐23 & 3‐24) that bind across the mutation locus. The product sizes of the ∆*fetC* mutants and wild type (WT) are 8.5 kb and 7.0 kb, respectively. (d) PCR screening for the ∆*sidA* mutants with two primers (LJNTF1‐05 & 1‐07) that bind across the mutation locus. The product sizes of the ∆*sidA* mutants and WT are 5.0 kb and 3.4 kb, respectively. (e) Vector map of the *fetC* gene replacement construct, pΔ*fetC*, which was used as a template to amplify split marker DNA fragments for strain Fl1 transformation. Feature representations: Primers (prefix = LJNTF) for amplifying and checking vector constituent DNA fragments and ligations (including pairs LJNTF1‐62 & LJNTF3‐9 and LJNTF1‐61 & LJNTF2‐77) and for screens of *fetC* mutants and complemented strains are shown. (f) Vector map of the *sidA* gene replacement construct, pΔ*sidA*, which was used as a template to amplify split marker DNA fragments for strain Fl1 transformation. In (a) and (b), genes are coloured with arrows, while coding sequences (CDSs) are given as grey segmented arrows. The flanking sequences for homologous recombination are shown as black rectangles.

**FIGURE S2.** Colony growth rates of *Epichloë festucae* under defined medium. (a) 50 μM FeCl_3_. (b) 100 μM FeCl_3_. Mean values of a single treatment (*n* = 6) are shown. ).

**FIGURE S3.** Morphology of the *Epichloë festucae* wild type (WT) and mutants after growth for 10 days on defined medium with different iron supply. Top and side views of the colony morphologies of the *E. festucae* WT and mutants (∆*fetC*, ∆*fetC*/*fetC*, ∆*sidA*, ∆*sidA*/*sidA*, and ∆*fetC*/∆*sidA*) after growth for 10 days at 23°C on defined medium with iron chelation (−Fe: 100 μM bathophenanthroline disulphonic acid [BPS]) or varied iron concentrations (+0Fe: 0 μM FeCl_3_, +25Fe: 25 μM FeCl_3_, +50Fe: 50 μM FeCl_3_, +100Fe: 100 μM FeCl_3_). Scale bar = 500 μm.

**FIGURE S4.** Hyphal morphology of *Epichloë festucae* hyphae after 1 week of growth on potato dextrose agar. Hyphae of *E. festucae* strains were stained with calcofluor white and then observed by confocal laser scanning microscopy. Representative hyphal tips and fusions are indicated by arrows and arrowheads, respectively. Scale bars = 20 μm.

**FIGURE S5.** Extracted liquid chromatography–mass spectrometry (LC‐MS) ion chromatograms for ferricrocin (*m*/*z* 771), ferriepichloënin A (FEA) [M + 2H]^2+^ (*m*/*z* 569), and FEA [M + H]^+^ (*m*/*z* 1136) in mycelium from 5‐day‐old iron‐depleted cultures of wild‐type *Epichloë festucae* Fl1 (WT), Δ*fetC*, Δ*fetC*/*fetC*, Δ*sidA*, Δ*sidA*/*sidA*, and Δ*fetC*/Δ*sidA*.

**FIGURE S6.** Extracted chromatography–mass spectrometry (LC‐MS) ion chromatograms for ferricrocin (*m*/*z* 771), ferriepichloënin A (FEA) [M + 2H]^2+^ (*m*/*z* 569), and FEA [M + H]^+^ (*m*/*z* 1136) in supernatant from 5‐day‐old iron‐depleted cultures of wild‐type *Epichloë festucae* Fl1 (WT), Δ*fetC*, Δ*fetC*/*fetC*, Δ*sidA*, Δ*sidA*/*sidA*, and Δ*fetC*/Δ*sidA*.

**FIGURE S7.** The flowering characteristics of plants infected with *Epichloë festucae* wild type (WT) and mutant derivatives. Boxplots illustrating (a) reproductive tiller percentage (%) in parent plants without *E. festucae* infection (E−) or infected with *E. festucae* WT, ∆*fetC*, ∆*fetC*/*fetC*, Δ*sidA*, Δ*sidA*/*sidA*, and Δ*fetC*/Δ*sidA*, (b) spike length (cm), (c) total spikelet number per spike, (d) fertile spikelet number per spike, and (e) fertile spikelet percentage (%) in parent plants without *E. festucae* infection (E−) or infected with *E. festucae* WT, ∆*fetC*, ∆*fetC*/*fetC*, ∆*sidA*, ∆*sidA*/*sidA*, and ∆*fetC*/∆*sidA*. Each blue dot represents one biological replicate.

**FIGURE S8.** Gene expression of *ftrA* in *Epichloë festucae* wild type (WT), ∆*fetC* strain G22, ∆*fetC* strain G24, and ∆*fetC*/*fetC* strains. The gene expression levels were calculated relative to the gene expression level in WT under iron‐depleted conditions.

**FIGURE S9.** *Epichloë festucae*‐infected ryegrass plants growing in hydroponic conditions with high iron and low iron supply. *E. festucae*‐infected ryegrass plants growing in hydroponic conditions with high iron (50 μM) and low iron supply (500 nM).

**FILE S1.** Supplementary Methods. Details of construction, validation, and selection of deletion mutants; gene expression quantification.

**TABLE S1.** Biological materials used in this study.

**TABLE S2.** Primers used in this study.

**TABLE S3.** Raw data for the figures presented in this study.

## Data Availability

Raw data that support the findings of this study are available in Table S3.
