# Supplementary figures and images for: High‐affinity iron uptake is required for optimal Epichloë festucae colonization of Lolium perenne and seed transmission
Source: Mol Plant Pathol. 2023 Jul 21;24(11):1430–42. doi: 10.1111/mpp.13379 (PMC10576175; doi:10.1111/mpp.13379)

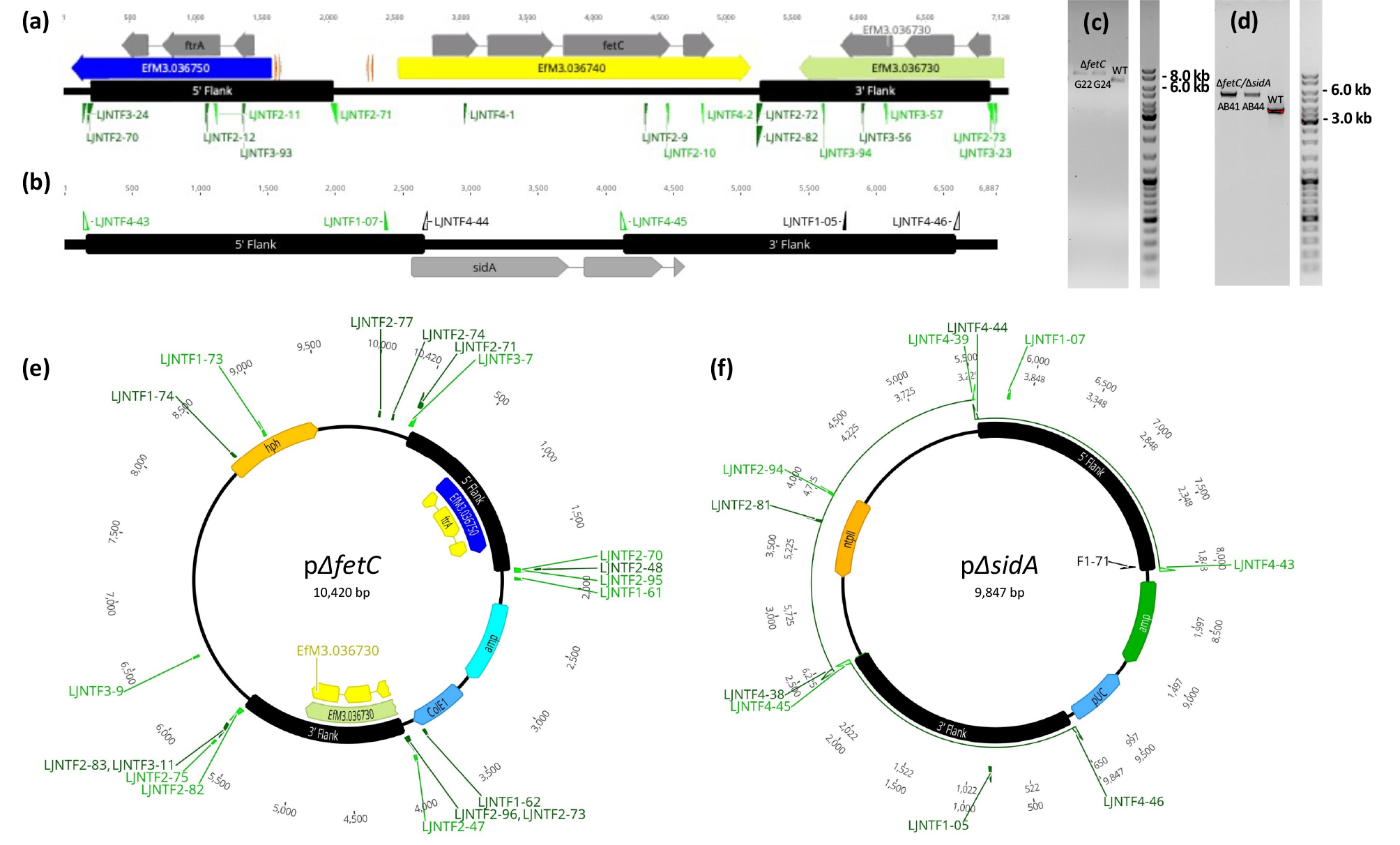

Supplement: Supplementary file 1 — FIGURE S1. Constructs of Epichloë festucae ΔfetC and ΔfetC/ΔsidA strains. (a) Gene replacement of fetC with the hph cassette by homologous recombination. Long coloured arrows represent the transcript for each gene model EfM3.036730‐50 and short red arrows denote potential SreA transcription factor binding sites. (b) Replacement of the sidA gene with the nptII gene by homologous recombination. (c) PCR screening for the ∆fetC mutants with two primers (LJNTF3‐23 & 3‐24) that bind across the mutation locus. The product sizes of the ∆fetC mutants and wild type (WT) are 8.5 kb and 7.0 kb, respectively. (d) PCR screening for the ∆sidA mutants with two primers (LJNTF1‐05 & 1‐07) that bind across the mutation locus. The product sizes of the ∆sidA mutants and WT are 5.0 kb and 3.4 kb, respectively. (e) Vector map of the fetC gene replacement construct, pΔfetC, which was used as a template to amplify split marker DNA fragments for strain Fl1 transformation. Feature representations: Primers (prefix = LJNTF) for amplifying and checking vector constituent DNA fragments and ligations (including pairs LJNTF1‐62 & LJNTF3‐9 and LJNTF1‐61 & LJNTF2‐77) and for screens of fetC mutants and complemented strains are shown. (f) Vector map of the sidA gene replacement construct, pΔsidA, which was used as a template to amplify split marker DNA fragments for strain Fl1 transformation. In (a) and (b), genes are coloured with arrows, while coding sequences (CDSs) are given as grey segmented arrows. The flanking sequences for homologous recombination are shown as black rectangles. [file MPP-24-1430-s007.tif]

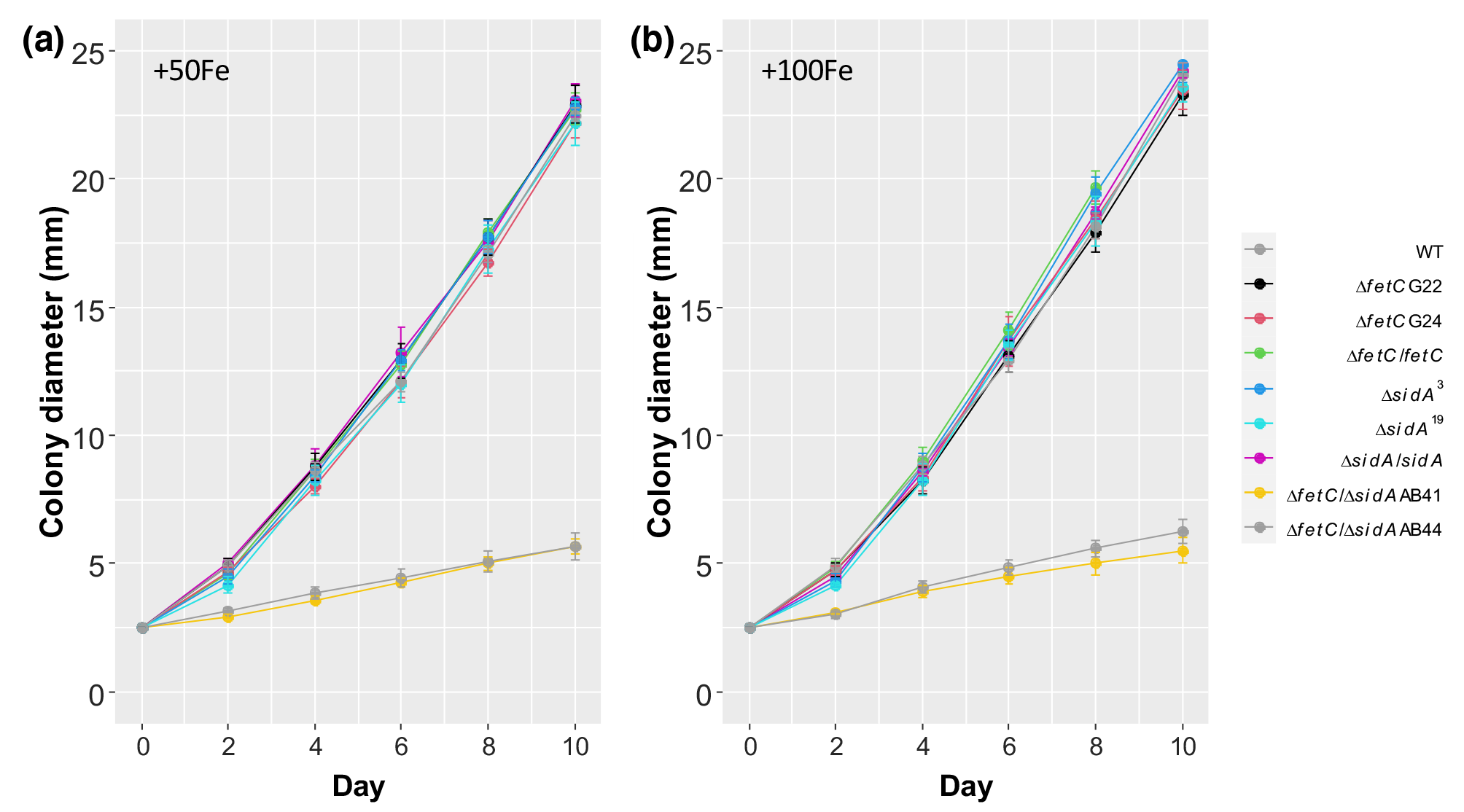

Supplement: Supplementary file 2 — FIGURE S2. Colony growth rates of Epichloë festucae under defined medium. (a) 50 μM FeCl3. (b) 100 μM FeCl3. Mean values of a single treatment (n = 6) are shown. ). [file MPP-24-1430-s005.tif]

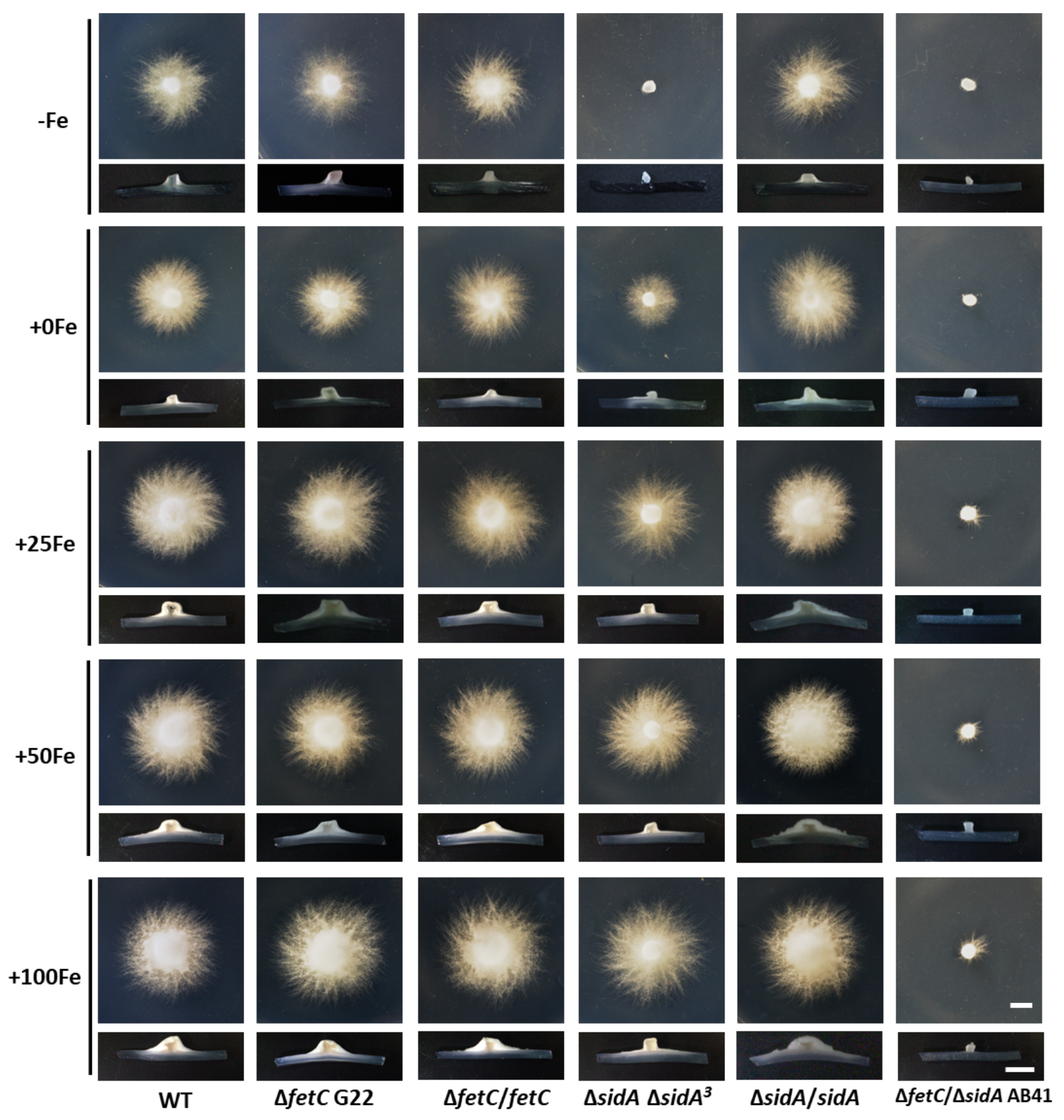

Supplement: Supplementary file 3 — FIGURE S3. Morphology of the Epichloë festucae wild type (WT) and mutants after growth for 10 days on defined medium with different iron supply. Top and side views of the colony morphologies of the E. festucae WT and mutants (∆fetC, ∆fetC/fetC, ∆sidA, ∆sidA/sidA, and ∆fetC/∆sidA) after growth for 10 days at 23°C on defined medium with iron chelation (−Fe: 100 μM bathophenanthroline disulphonic acid [BPS]) or varied iron concentrations (+0Fe: 0 μM FeCl3, +25Fe: 25 μM FeCl3, +50Fe: 50 μM FeCl3, +100Fe: 100 μM FeCl3). Scale bar = 500 μm. [file MPP-24-1430-s012.tif]

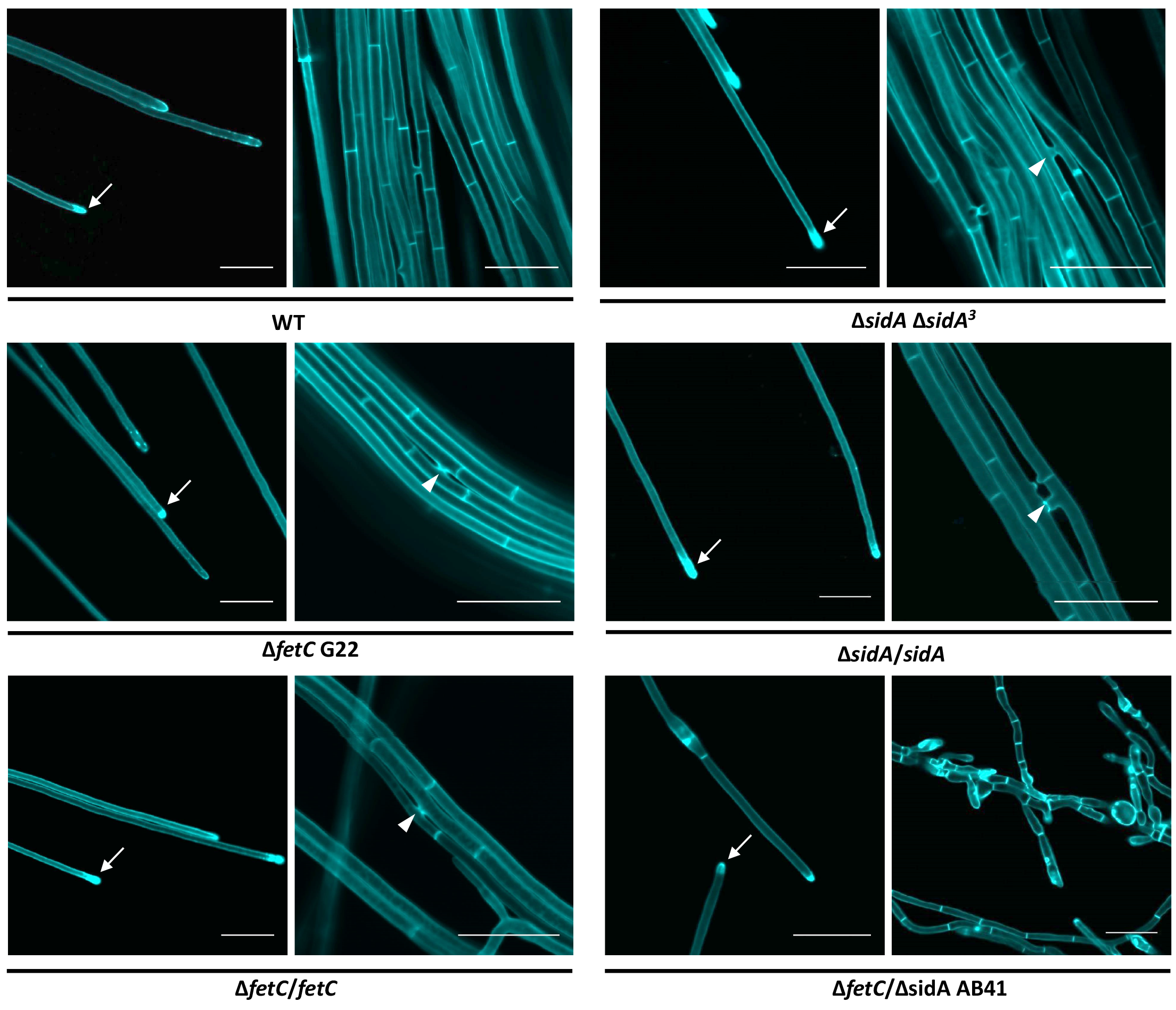

Supplement: Supplementary file 4 — FIGURE S4. Hyphal morphology of Epichloë festucae hyphae after 1 week of growth on potato dextrose agar. Hyphae of E. festucae strains were stained with calcofluor white and then observed by confocal laser scanning microscopy. Representative hyphal tips and fusions are indicated by arrows and arrowheads, respectively. Scale bars = 20 μm. [file MPP-24-1430-s008.tif]

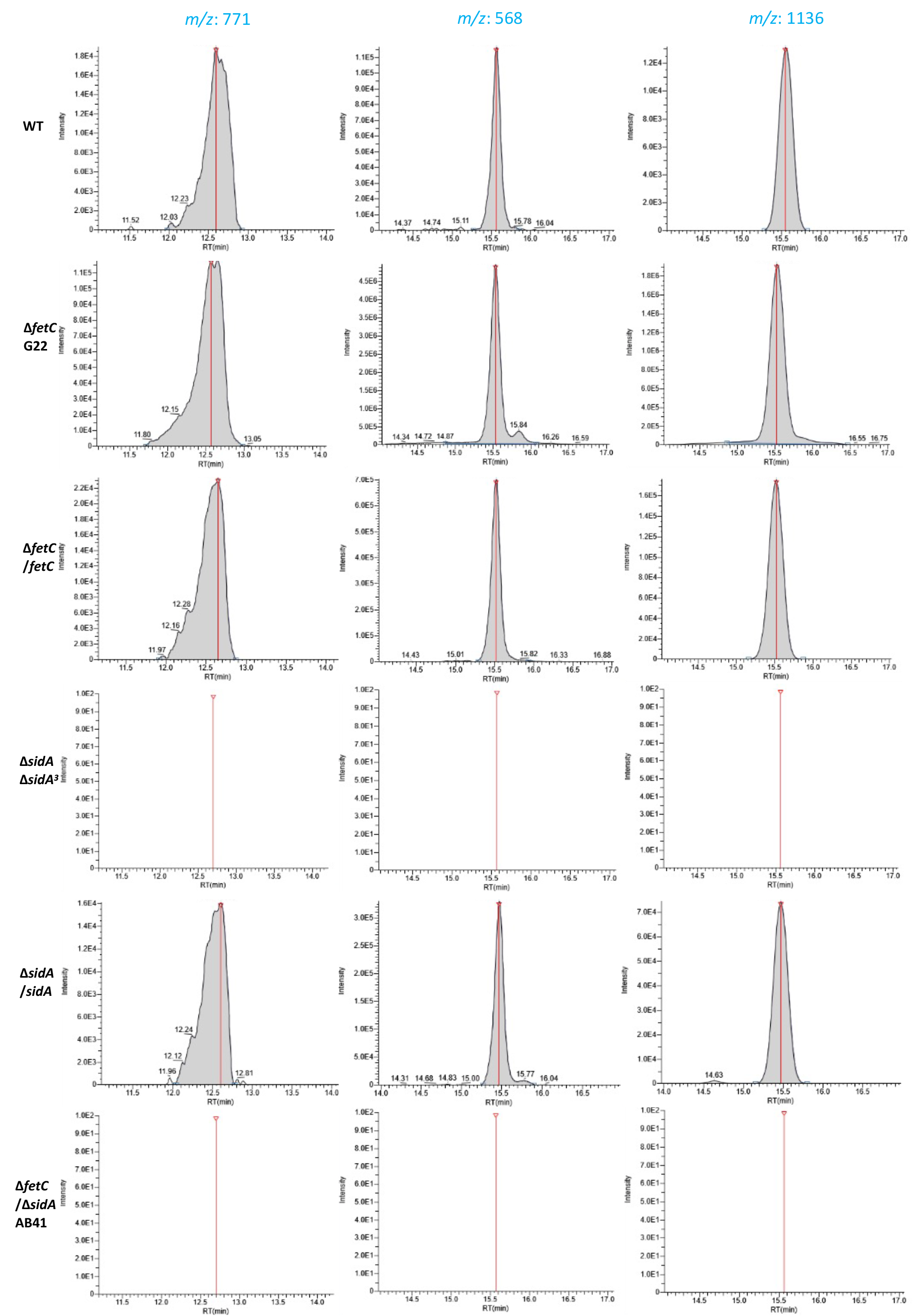

Supplement: Supplementary file 5 — FIGURE S5. Extracted liquid chromatography–mass spectrometry (LC‐MS) ion chromatograms for ferricrocin (m/z 771), ferriepichloënin A (FEA) [M + 2H]2+ (m/z 569), and FEA [M + H]+ (m/z 1136) in mycelium from 5‐day‐old iron‐depleted cultures of wild‐type Epichloë festucae Fl1 (WT), ΔfetC, ΔfetC/fetC, ΔsidA, ΔsidA/sidA, and ΔfetC/ΔsidA. [file MPP-24-1430-s013.tif]

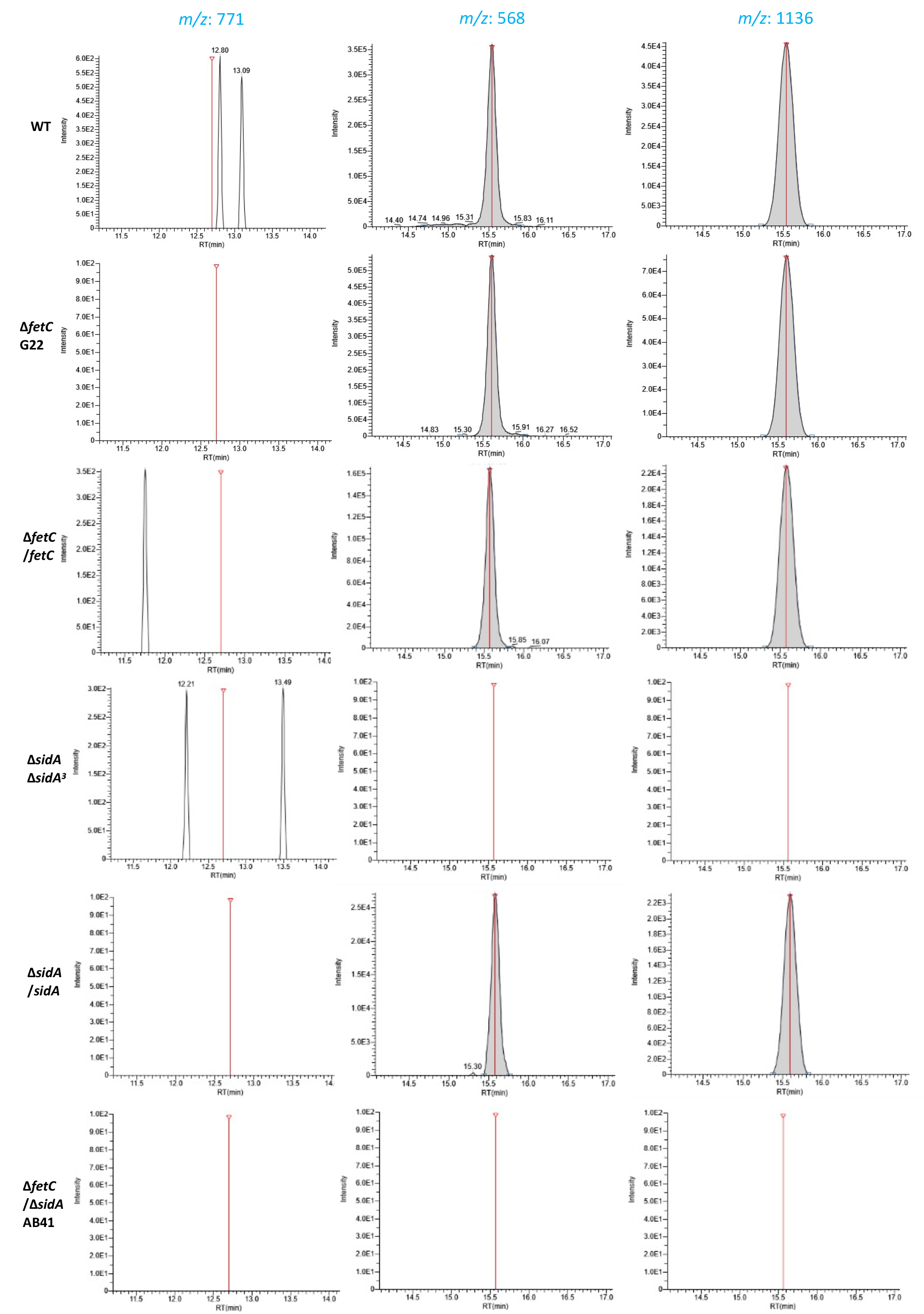

Supplement: Supplementary file 6 — FIGURE S6. Extracted chromatography–mass spectrometry (LC‐MS) ion chromatograms for ferricrocin (m/z 771), ferriepichloënin A (FEA) [M + 2H]2+ (m/z 569), and FEA [M + H]+ (m/z 1136) in supernatant from 5‐day‐old iron‐depleted cultures of wild‐type Epichloë festucae Fl1 (WT), ΔfetC, ΔfetC/fetC, ΔsidA, ΔsidA/sidA, and ΔfetC/ΔsidA. [file MPP-24-1430-s006.tif]

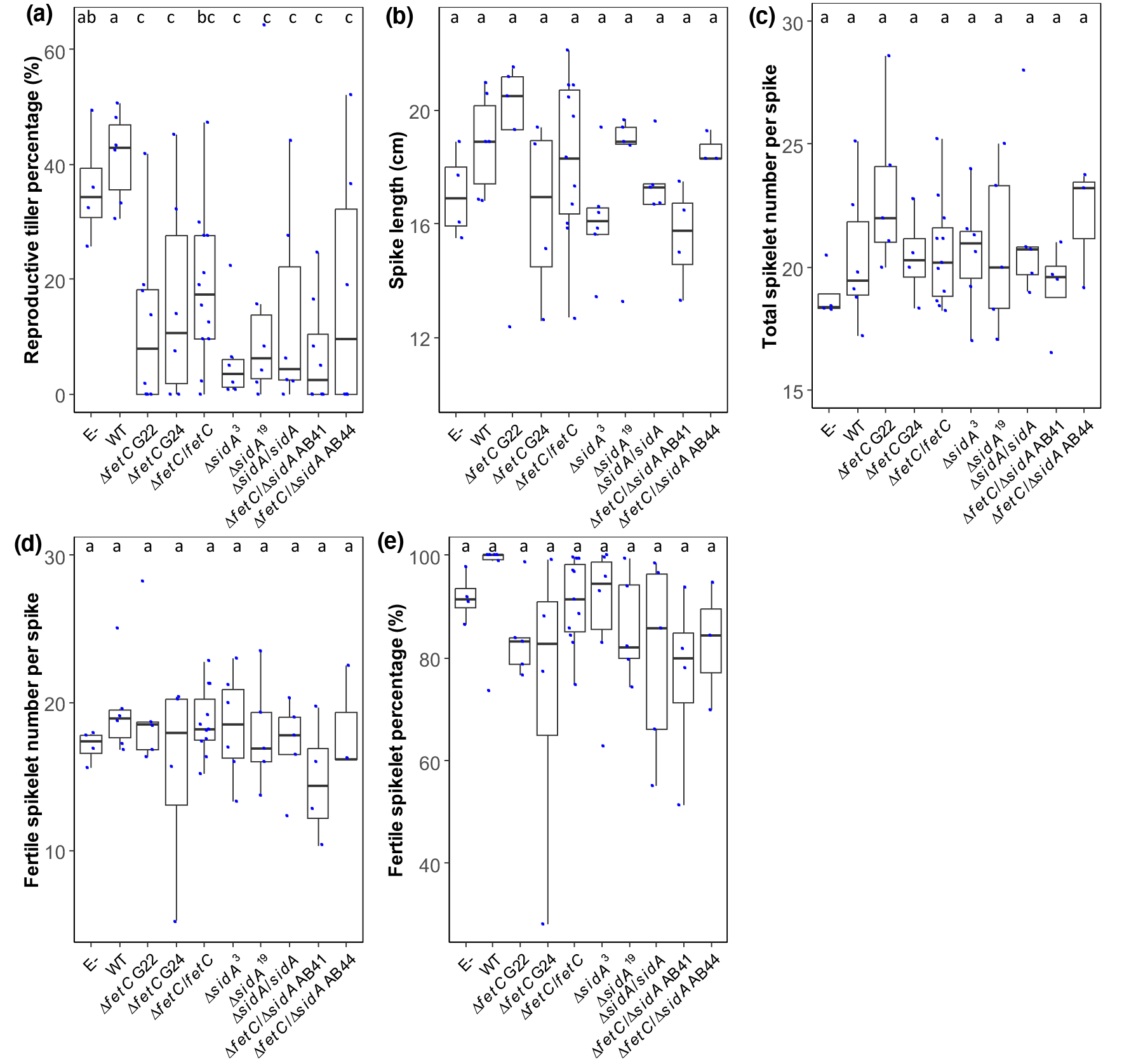

Supplement: Supplementary file 7 — FIGURE S7. The flowering characteristics of plants infected with Epichloë festucae wild type (WT) and mutant derivatives. Boxplots illustrating (a) reproductive tiller percentage (%) in parent plants without E. festucae infection (E−) or infected with E. festucae WT, ∆fetC, ∆fetC/fetC, ΔsidA, ΔsidA/sidA, and ΔfetC/ΔsidA, (b) spike length (cm), (c) total spikelet number per spike, (d) fertile spikelet number per spike, and (e) fertile spikelet percentage (%) in parent plants without E. festucae infection (E−) or infected with E. festucae WT, ∆fetC, ∆fetC/fetC, ∆sidA, ∆sidA/sidA, and ∆fetC/∆sidA. Each blue dot represents one biological replicate. [file MPP-24-1430-s003.tif]

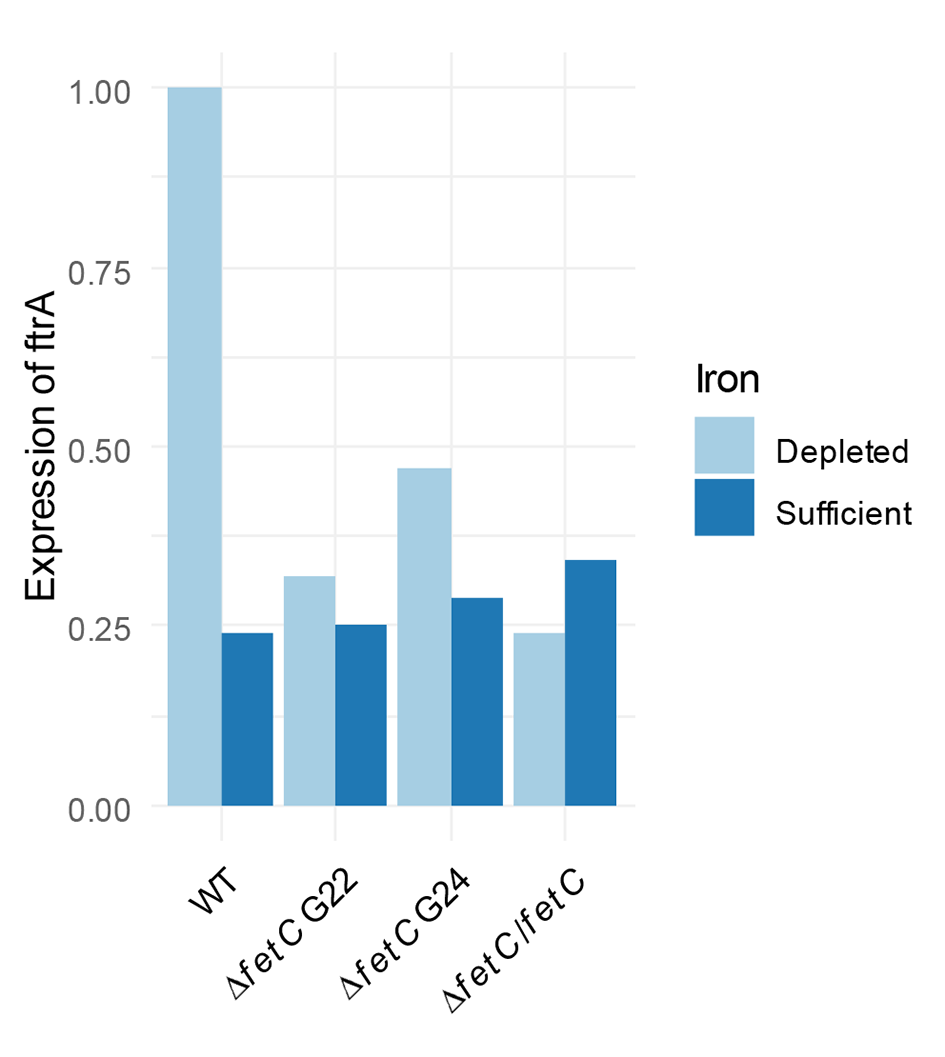

Supplement: Supplementary file 8 — FIGURE S8. Gene expression of ftrA in Epichloë festucae wild type (WT), ∆fetC strain G22, ∆fetC strain G24, and ∆fetC/fetC strains. The gene expression levels were calculated relative to the gene expression level in WT under iron‐depleted conditions. [file MPP-24-1430-s011.tif]

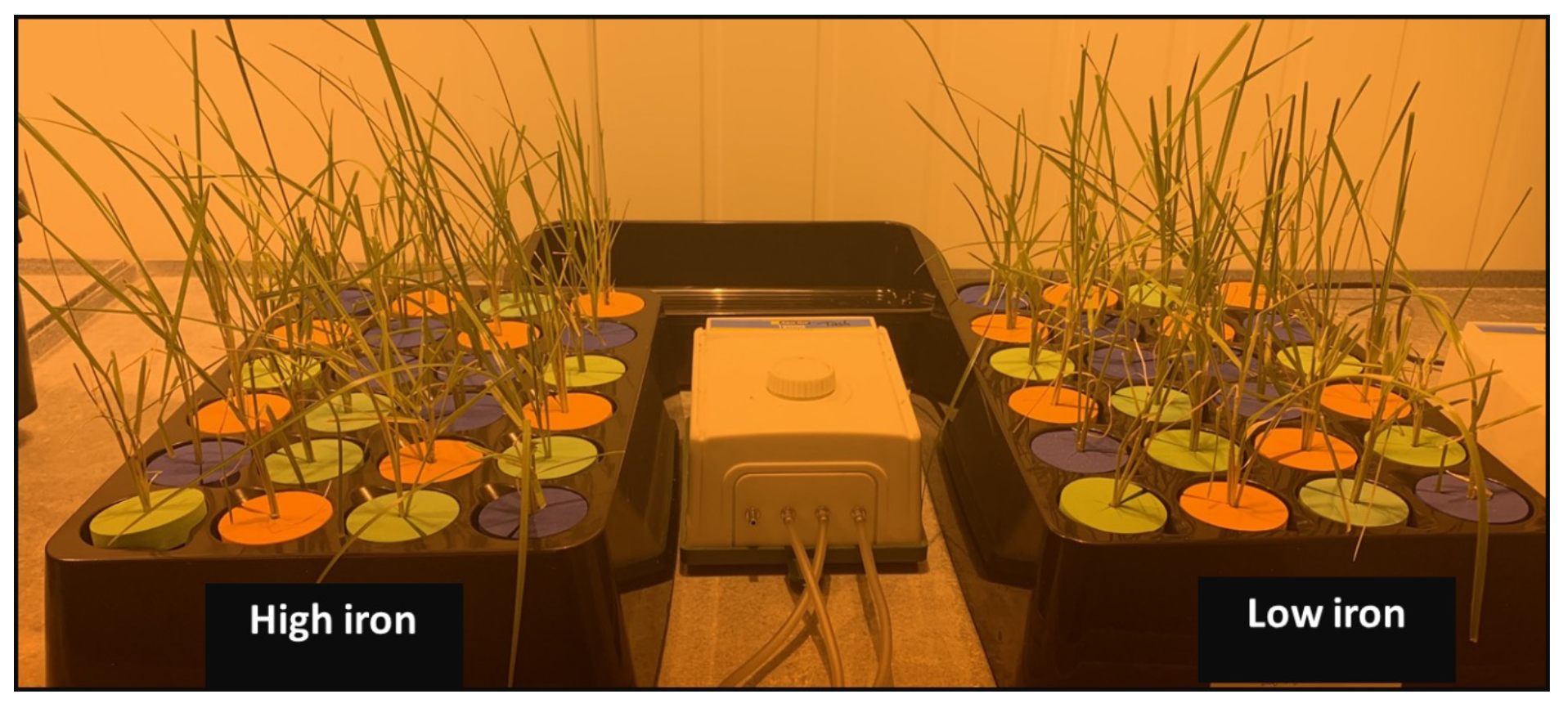

Supplement: Supplementary file 9 — FIGURE S9. Epichloë festucae‐infected ryegrass plants growing in hydroponic conditions with high iron and low iron supply. E. festucae‐infected ryegrass plants growing in hydroponic conditions with high iron (50 μM) and low iron supply (500 nM). [file MPP-24-1430-s001.tif]
