# Supplementary material for: High‐affinity iron uptake is required for optimal Epichloë festucae colonization of Lolium perenne and seed transmission
Source: Mol Plant Pathol. 2023 Jul 21;24(11):1430–42. doi: 10.1111/mpp.13379 (PMC10576175; doi:10.1111/mpp.13379)
Supplement: Supplementary file 10 — FILE S1. Supplementary Methods. Details of construction, validation, and selection of deletion mutants; gene expression quantification. [file MPP-24-1430-s009.docx]

**Supplementary Methods**

**Details of construction, validation and selection of deletion mutants**

Table S2 contains a list of the primers used in this study. The amplification of cloning products used PrimerSTAR HS DNA polymerase (Takara Bio Inc., Japan), under the manufacturer’s conditions and using a touch-down PCR programme: Cycle 1 - 1 × 94°C for 30 s; Cycle 2 - 8 × (98°C for 10 s, *Tm - 0.5°C per cycle for 7 s, 68°C for 1 min per kb); Cycle 3 - 25 × (98°C for 10 s, 62°C for 7 s, 72°C for 1-2 min per kb); Cycle 4 - 1 × (98°C for 10 s, 68°C for 2 min per kb), where *Tm is the primer annealing temperature. The DNA fragments for transformation were purified either by agarose gel purification using the Zymo Zymoclean™ gel DNA recovery kit (Zymo Research Corp., Orange, USA) or by DNA Clean & Concentrator-5 columns (Zymo Research Corp.). Purified PCR products were used for subcloning in *Escherichia coli* (Top10, Invitrogen, Carlsbad, USA) as per established methods or to transform *E. festucae* protoplasts (Young et al., 1998). Plasmids were checked for correct status using restriction enzyme analysis, PCR and/or DNA sequencing using universal M13 primers (Massey Genome Service, Palmerston North, New Zealand). Transformation of *E. festucae* cells was achieved via homologous recombination following protoplast-mediated transformation (Johnson et al., 2013). Recombinants were selected with hygromycin (150 µg/mL) or geneticin (200 μg/mL) on regeneration medium and resistant colonies were nuclear purified by three rounds of subculture (Young et al., 2005). Approximately 100 colonies were checked and verified as *E. festucae* mutants or complemented strains using a combination of PCR, diagnostic for each construction that were created as follows.

The *fetC* gene disruption strain was generated through a Gibson cloning method (Gibson et al., 2009), followed by a split marker transformation approach (Rahnama et al., 2017) that replaced *fetC* of the WT strain with a gene cassette (*hph*) conferring hygromycin resistance (Figure S1). Briefly, *hph* was amplified from plasmid pDONR221-Hyg (Fleetwood et al., 2007), and the 5′ and 3′ flanking regions of the *fetC* gene from genomic DNA of the WT strain. The three purified PCR products were assembled with a PCR-amplified vector backbone of plasmid pII99 (Namiki et al., 2001) using the NEBuilder assembly reaction (New England Biolabs Inc., Ipswich, USA). Using the resulting verified plasmid pΔ*fetC* as a template, two overlapping split marker fragments, Hyg-Δ*fetC* 5′ and Hyg-Δ*fetC* 3′, were generated by PCR and 100 fmol of purified fragments were used to transform *E. festucae* WT protoplasts.

Δ*fetC*/Δ*sidA* double mutants were derived from verified Δ*fetC* mutants, using Golden Gate cloning (Hartley et al., 2000), followed by split marker transformation to substitute *E. festucae* *sidA* for a gene cassette *nptII* encoding for geneticin resistance (Namiki et al. 2001) (Figure S1). The *nptII* cassette was amplified from plasmid pII99, and the 5′ and 3′ flanking regions of the *sidA* gene from gDNA of the WT strain; the primers each designed with sequence compatible with the AarI type-IIS restriction system. Each of the three gel purified PCR products were individually subcloned into a Zero Blunt™ TOPO™ vector (Life Technologies, Carlsbad, USA) and following DNA sequencing, the three plasmids were combined in a AarI restriction enzyme and ligation reaction with a recipient vector, pType-IIs (ThermoFisher, Waltham, USA) as per manufacturer’s instructions. Following *E. coli* transformation and selection with ampicillin (50 μg/mL), the resulting plasmid pΔ*sidA* was used as a template to generate two split marker fragments by PCR, while Gen-Δ*sidA* 5′ and Gen-Δ*sidA* 3′ were used for transforming *E. festucae* Δ*fetC* protoplasts.

Using a real-time PCR (qPCR) method, the Δ*fetC* and Δ*fetC*/Δ*sidA* mutants were validated for a single-locus insertion as described by Solomon & Hane (2008) using NRPS1 as a single copy reference gene (Rasmussen et al., 2007), with the target genes for the mutants being *hph* for Δ*fetC* and *nptII* for Δ*fetC*/Δ*sidA*, using gene-specific primers. The reference sample for measuring gene copy number is an *E. festucae* ∆*sidN* mutant with a single-copy *hph* gene that has been genome-sequenced. In 96-well plates, qPCR was done using a LightCycler 480 real-time PCR apparatus (Roche Diagnostics, Mannheim, Germany). The qPCR reaction was carried out in a 10 μL reaction mixture including 5 μL KAPA SYBR® FAST qPCR Master Mix (2X) (Kapa Biosystems, Woburn, USA), 0.2 μM each of the forward and reverse primers (10 μM), and 2-6 ng *Epichloë* DNA. Endophyte DNA was extracted using the Quick-DNA™ Fungal/Bacterial Miniprep Kit (Zymo Research Corp.) from mycelium cultured on cellophane membranes covering PDA for 3 days. The PCR conditions were 95°C for 3 min, followed by 45 cycles of 95°C for 10 s and 60°C for 20 s, plus a final amplification at 72°C for 5 s. Each PCR reaction was performed three times. In this work, only transformants with verified single-site substitutions were employed for study.

Two independent Δ*fetC* mutants (G22 and G24) and two Δ*fetC*/Δ*sidA* mutants developed from each of the Δ*fetC* transformants (AB41, AB44), respectively were chosen. Both Δ*fetC* and Δ*fetC*/Δ*sidA* were disrupted at similar frequencies (~10%). For gene complementation of the mutated *fetC* gene, Δ*fetC* mutants (G22) protoplasts were co-transformed with a 4.4 kb PCR product (using primers LJNTF3-93 & LJNTF3-94) containing an intact *fetC* expression cassette from strain Fl1 and plasmid, pII99 (carrying the *nptII* gene), which confers resistance to geneticin (200 µg/mL). One Δ*fetC*/*fetC* rescued strain was selected for study. Additionally, previously generated Δ*sidA* mutants (Δ*sidA*^3^, Δ*sidA*^19^) and Δ*sidA*/*sidA* (complemented from Δ*sidA*^19^), were used as controls (Forester et al., 2018). The two independently generated colonies of each mutant were used throughout this study.

***Gene expression quantification***

Gene expression of *E. festucae ftrA*, encoding a high affinity iron permease from *E. festucae* was quantified from WT, ∆*fetC* strain G22, ∆*fetC* strain G24 and ∆*fetC*/*fetC*. Mycelium was harvested from fungal cultures grown on solid defined medium with two iron concentrations for 10 days, i.e., iron-depleted (defined medium with 100 µM BPS) and iron-sufficient (defined medium with 50 µM FeCl_3_). Fungal RNA was extracted by using a combined TRIzol™ (Thermo Fisher Scientific Inc., Waltham, USA) and chloroform, followed by a further cleanup with the RNeasy MinElute Cleanup Kit (Qiagen, Hilden, Germany). cDNA was synthesized using QuantiTect Reverse Transcription Kit (Qiagen) with 1 µg DNase-treated total RNA (VILO cDNA synthesis kit, Invitrogen). RT-qPCR was performed in a LightCycler® 480 Instrument using KAPA SYBR® FAST qPCR reagents (KAPA Biosystems, Boston, USA) according to manufacturer’s protocol in a 10 µL reaction containing 0.25 µM of each primer pair (Table S2). Two endophyte housekeeping genes (a 60S ribosomal protein L35 and gamma actin, Table S2) were used to normalize expression levels.

Fleetwood, D.J., Scott, B., Lane, G.A., Tanaka, A. & Johnson, R.D. (2007) A complex ergovaline gene cluster in *Epichloë* endophytes of grasses. *Applied and Environmental Microbiology*, 73, 2571–2579.

Forester, N.T., Lane, G.A., Steringa, M., Lamont, I.L. & Johnson, L.J. (2018) Contrasting roles of fungal siderophores in maintaining iron homeostasis in *Epichloë festucae*. *Fungal Genetics and Biology*, 111, 60–72.

Gibson, D.G., Young, L., Chuang, R.-Y., Venter, J.C., Hutchison, C.A. & Smith, H.O. (2009) Enzymatic assembly of DNA molecules up to several hundred kilobases. *Nature Methods*, 6, 343–345.

Hartley, J.L., Temple, G.F. & Brasch, M.A. (2000) DNA cloning using in vitro site-specific recombination. *Genome Research*, 10, 1788–1795.

Johnson, L.J., Koulman, A., Christensen, M., Lane, G.A., Fraser, K., Forester, N., et al. (2013) An extracellular siderophore is required to maintain the mutualistic interaction of *Epichloë festucae* with *Lolium perenne*. *PLoS Pathogens*, 9, e1003332.

Namiki, F., Matsunaga, M., Okuda, M., Inoue, I., Nishi, K., Fujita, Y., et al. (2001) Mutation of an arginine biosynthesis gene causes reduced pathogenicity in *Fusarium oxysporum* f. sp. *melonis*. *Molecular Plant-Microbe Interactions*, 14, 580–584.

Rahnama, M., Forester, N., Ariyawansa, K., Voisey, C.R., Johnson, L.J., Johnson, R.D., et al. (2017) Efficient targeted mutagenesis in *Epichloë festucae* using a split marker system. *Journal of Microbiological Methods*, 134, 62–65.

Rasmussen, S., Parsons, A.J., Bassett, S., Christensen, M.J., Hume, D.E., Johnson, L.J., et al. (2007) High nitrogen supply and carbohydrate content reduce fungal endophyte and alkaloid concentration in *Lolium perenne*. *New Phytologist*, 173, 787–797.

Solomon, E.I., Sundaram, U.M. & Machonkin, T.E. (1996) Multicopper oxidases and oxygenases. *Chemical Reviews*, 96, 2563–2605.

Young, C., Itoh, Y., Johnson, R., Garthwaite, I., Miles, C.O., Munday-Finch, S.C., et al. (1998) Paxilline-negative mutants of *Penicillium paxilli* generated by heterologous and homologous plasmid integration. *Current Genetics*, 33, 368–377.

Young, C.A., Bryant, M.K., Christensen, M.J., Tapper, B.A., Bryan, G.T. & Scott, B. (2005) Molecular cloning and genetic analysis of a symbiosis-expressed gene cluster for lolitrem biosynthesis from a mutualistic endophyte of perennial ryegrass. *Molecular Genetics and Genomics*, 274, 13–29.
