# Supplementary material for: High‐affinity iron uptake is required for optimal Epichloë festucae colonization of Lolium perenne and seed transmission
Source: Mol Plant Pathol. 2023 Jul 21;24(11):1430–42. doi: 10.1111/mpp.13379 (PMC10576175; doi:10.1111/mpp.13379)
Supplement: Supplementary file 11 — TABLE S1. Biological materials used in this study. [file MPP-24-1430-s010.docx]

Table S1. Biological materials used in this study

| *E. festucae strains* | Independent  isolates | Description | Plasmid(s)/construct | Source or Reference | |
| --- | --- | --- | --- | --- | --- |
| Fl1 | a | *E. festucae* Fl1 isolated from a healthy infected *L. perenne* plant | nil | This study | |
| Fl1 (WT) | 1, 2, 3 | Protoplast regenerants of Fl1 ‘a’; used as independent controls | nil | This study | |
| Δ*sidA* | Δ*sidA*^3^, Δ*sidA*^19^ | Δ*sidA::PgpdA-hph;* Hyg^R^ | p∆*sidA* | Forester et al., 2018 | |
| Δ*sidA*/*sidA* | Complemented from Δ*sidA*^19^ | Δ*sidA::PgpdA-hph*/1G18/*nptII*; Hyg^R^*,* Gen^R^ | p∆*sidA*; fosmid 1G18 + p*II99* | Forester et al., 2018 | |
| Δ*fetC* | G22, G24 | Δ*fetC::PgpdA-hph;* Hyg^R^ | p∆*fetC* | This study | |
| Δ*fetC*/*fetC* | Complemented from G22 | Δ*fetC::PgpdA-hph*/*fetC*/*nptII*; Hyg^R^*,* Gen^R^ | p∆*fetC*; *fetC* (PCR) + p*II99* | This study | |
| Δ*fetC*/Δ*sidA* | AB41, AB44 | Δ*fetC::PgpdA-hph*, *ΔsidA:: PgpdA-nptII*; Hyg^R^*,* Gen^R^ | p∆*fetC* + p∆*sidA* | This study | |
| Hyg^R^ = hygromycin resistant, Gen^R^ = geneticin resistant. | | | | |  |

Forester, N.T., Lane, G.A., Steringa, M., Lamont, I.L. & Johnson, L.J. (2018) Contrasting roles of fungal siderophores in maintaining iron homeostasis in *Epichloë festucae*. *Fungal Genetics and Biology*, 111, 60–72.
