# Supplementary material for: High‐affinity iron uptake is required for optimal Epichloë festucae colonization of Lolium perenne and seed transmission
Source: Mol Plant Pathol. 2023 Jul 21;24(11):1430–42. doi: 10.1111/mpp.13379 (PMC10576175; doi:10.1111/mpp.13379)
Supplement: Supplementary file 12 — TABLE S2. Primers used in this study. [file MPP-24-1430-s004.docx]

### Table S2. Primers used in this study.

| Code | Sequence (5’ to 3’) | bp | Purpose | Alias |  |
| --- | --- | --- | --- | --- | --- |
| LJNTF4-43 | CACCACACACCTGCGACTCTGGGACACCGACAACGTG | 22+15 | cloning | sidA 5’KO_F |  |
| LJNTF4-44 | CACCACACACCTGCGACTAGGTGCTGCCGATGCTGTTCAG | 22+18 | cloning | sidA 5’KO_R |  |
| LJNTF4-45 | CACCACACACCTGCGACTGACCAGATGCCACTGACGAGGA | 22+18 | cloning | sidA 3’KO_F |  |
| LJNTF4-46 | CACCACACACCTGCGACTATGCGAAGTAGAGGTGGAAGTG | 22+18 | cloning | sidA 3’KO_R |  |
| LJNTF4-39 | CACCACACACCTGCGACTACCTGAGGCGAGGAAGCGGAAGAG | 22+20 | cloning | Gen^R^_F |  |
| LJNTF4-38 | CACCACACACCTGCGACTGGTCGCGGCTACAATTAATACATAACC | 22+23 | cloning | Gen^R^_R |  |
| LJNTF2-81 | TCGGCAGGAGCAAGGTGAGA | 20 | split marker | pSML-∆*sidA* 5′_F |  |
| LJNTF4-43 | CACCACACACCTGCGACTCTGGGACACCGACAACGTG | 22+15 | split marker | pSML-∆*sidA* 5′_R |  |
| LJNTF4-46 | CACCACACACCTGCGACTATGCGAAGTAGAGGTGGAAGTG | 22+18 | split marker | pSMR-Δ*sidA* 3′_F |  |
| LJNTF2-94 | TGACTGGGCACAACAGACAA | 20 | split marker | pSMR-Δ*sidA* 3′_R |  |
| LJNTF2-70 | TAACTATGCGGCATCAGAGCAGCTTGAATCGCATGGTGCAGA | 22+20 | cloning | fetC 5’KO_F |  |
| LJNTF2-71 | ACATGGTCATAGCTGTTTCCTGGCGGACTTGCCTATCGTGTAAT | 24+20 | cloning | fetC 5’KO_R |  |
| LJNTF2-82 | TCCAGTCGGGAAACCTGTCGTGTATCCCACCCACCCAATGAG | 22+20 | cloning | fetC 3’KO_F |  |
| LJNTF2-73 | CCTTTGAGTGAGCTGATACCGCTAACCGCCAAACCCGACGAA | 21+21 | cloning | fetC 3’KO_R |  |
| LJNTF3-11 | CACGACAGGTTTCCCGACTGGTATGAGTGCAGTGA | 22+13 | cloning | Hyg^R^ _F |  |
| LJNTF3-07 | GCCAGGAAACAGCTATGACCATGTAATACGACTCA | 24+11 | cloning | Hyg^R^_R |  |
| LJNTF2-47 | GCGGTATCAGCTCACTCAAA | 20 | cloning | pII99 backbone_F |  |
| LJNTF2-48 | CTGCTCTGATGCCGCATAGT | 20 | cloning | pII99 backbone_R |  |
| LJNTF1-73 | GTCCATCACAGTTTGCCAGT | 20 | split marker | pSML-∆*fetC* 5’_F |  |
| LJNTF2-96 | CGCCTTTGAGTGAGCTGATA | 20 | split marker | pSML-∆*fetC* 5’_R |  |
| LJNTF2-95 | GGCTTAACTATGCGGCATCAGA | 22 | split marker | pSMR-∆*fetC* 3’_F |  |
| LJNTF1-74 | CGATGTAGGAGGGCGTGGAT | 20 | split marker | pSMR-∆*fetC* 3’_R |  |
| LJNTF3-93 | CGAGACAGGCAGCACTACA | 19 | cloning | ∆*fetC* complement_F |  |
| LJNTF3-94 | CGAGCAGCACCGACACTA | 18 | cloning | ∆*fetC* complement_R |  |
| LJNTF3-24 | CACAGAAGGATCTGCCGTCT | 20 | PCR screening | fetC_5’ genome_F |  |
| LJNTF3-23 | TTCGGATTCCCGTGCTCGTT | 20 | PCR screening | fetC_3’ genome_R |  |
| LJNTF1-07 | GCATCTACTCTACTTCGTTTC | 21 | PCR screening | sidA_5' genome_F |  |
| LJNTF1-05 | TATCGCTGAATCCCACCATC | 20 | PCR screening | sidA_3' genome_R |  |
| LJNTF1‐62 | TTTCGCCACCTCTGACTTGA | 20 | PCR screening | p∆fetC_3’_F |  |
| LJNTF3‐9 | CGCCCATGAACTGGCTCTT | 19 | PCR screening | p∆fetC_3’_R |  |
| LJNTF1‐61 | TTGTCTGTAAGCGGATGCC | 19 | PCR screening | p∆fetC_5’_R |  |
| LJNTF2‐77 | TGCCTAGTGAATGCTCCGTA | 20 | PCR screening | p∆fetC_5’_F |  |
| LJNTF2-81 | TCGGCAGGAGCAAGGTGAGA | 20 | qPCR | Gen^R^_qPCR_F |  |
| LJNTF2-94 | TGACTGGGCACAACAGACAA | 20 | qPCR | Gen^R^_qPCR_R |  |
| LJNTF1-74 | CGATGTAGGAGGGCGTGGAT | 20 | qPCR | Hyg^R^_qPCR_F |  |
| LJNTF1-73 | GTCCATCACAGTTTGCCAGT | 20 | qPCR | Hyg^R^_qPCR_R |  |
| LJNTF3-97 | GTCCGATCATTCCAAGCTCGTT | 20 | qPCR | NRPS1_ qPCR_F |  |
| LJNTF3-98 | TGGTGGGAAGTTCCCTGCAC | 20 | qPCR | NRPS1_ qPCR_R |  |
| LJNTF2-25 | CTCTCGACCTCCGTGTCAAG | 20 | RT-qPCR | L35 qPCR_F |  |
| LJNTF2-26 | GAAGTGAGTGGTGCGCTTC | 19 | RT-qPCR | L35 qPCR_R |  |
| LJNTF2-27 | AAGTGTGATGTCGATGTCCG | 20 | RT-qPCR | gamma actin qPCR_F |  |
| LJNTF2-28 | TGCATACGGTCGGAGAGAC | 19 | RT-qPCR | gamma actin qPCR_R |  |
| LJNTF2-11 | CTTGGTCTGCATGATTGTCG | 20 | RT-qPCR | ftrA_F |  |
| LJNTF2-12 | TAATTCTCGCTGTTCTCCCA | 20 | RT-qPCR | ftrA_R |  |
| Abbreviations: bp, base pairs.  The underlined sequence in the "Sequence" column denotes the overhang sequence, and the underlined number in the "bp" column denotes the number of base pairs associated with it. | | | | | |
